# Prospective randomized controlled trial comparing the effect of Monocryl versus nylon sutures on patient- and observer-assessed outcomes following carpal tunnel surgery

**DOI:** 10.1177/17531934231178383

**Published:** 2023-06-09

**Authors:** Edward Wu, Robert Allen, Christopher Bayne, Robert Szabo

**Affiliations:** 1 Department of Orthopedic Surgery, UC Davis School of Medicine, Sacramento, CA, USA; 2 Department of Orthopedic Surgery, University of Minnesota Medical School, Minneapolis, MN, USA

**Keywords:** Carpal tunnel, Monocryl, POSAS, scar, randomized controlled trial, suture

## Abstract

Controversy remains regarding the optimal technique and suture type for wound closure after carpal tunnel surgery. Adult patients undergoing open carpal tunnel release were prospectively randomized to receive either interrupted, buried Monocryl sutures or traditional nylon horizontal mattress sutures for their wound closures. At the 2-week and 6-week postoperative visits, Patient and Observer Scar Assessment Scale questionnaires were completed. At 2 weeks, patients and observers had a significantly better opinion of incisions closed with Monocryl. By 6 weeks, neither patients nor observers found a difference between suture types in any category. Scars of wounds closed with Monocryl did not change appreciably in appearance between 2 and 6 weeks. However, patients and observers noted significant improvement in scar appearance in the nylon group over time. Monocryl suture represents an effective method for carpal tunnel closure that leads to improved patient- and observer-reported outcome scores in the early postoperative period compared with nylon.

**Level of evidence:** II

## Introduction

Carpal tunnel syndrome is a constellation of symptoms that occurs due to chronic compression of the median nerve within the fibro-osseous tunnel at the wrist. Over time, symptoms may progress from paraesthesia to permanent numbness and in severe cases, atrophy of the thenar musculature. Carpal tunnel decompression remains the most reliable and definitive treatment and is the most commonly performed elective procedure in the hand (Wildin et al., 2006). Since the introduction of endoscopic carpal tunnel release, attention has been given to scar tenderness as an important outcome variable of surgery. Controversy remains regarding which wound closure method optimizes wound healing, appearance and patient satisfaction in open carpal tunnel release.

The ideal method of wound closure following open carpal tunnel release would provide adequate strength during the proliferative wound healing period, cause minimal inflammatory reaction, require minimal postoperative care or clinic visits and produce a good cosmetic outcome with high patient satisfaction (Dosani et al., 2013; Theopold et al., 2012). Surgical site complications, such as dehiscence, inflammation, pain and infection, can markedly impair a patient’s hand function and quality of life. Therefore, it is important to know if the choice of suture material used for skin closure can affect outcomes, reduce adverse events and reduce costs.

Prior research evaluating the superiority of absorbable or non-absorbable sutures for wound closure in carpal tunnel surgery is largely inconclusive, with low quality of evidence and high risk of bias in previous comparison trials (Wade et al., 2018). Here we report the results of a rigorously conducted, prospective randomized controlled trial comparing the outcome of Monocryl (poliglecaprone 25, Ethicon, Inc., Raritan, NJ, USA) versus traditional nylon (Ethilon, Ethicon, Inc., Raritan, NJ, USA) sutures on scarring following carpal tunnel release. We chose to compare interrupted, horizontal mattress closure with nylon sutures and interrupted, buried, deep dermal closure with Monocryl because both are widely used techniques in hand surgery across institutions and were the preferred techniques by the three primary surgeons conducting the study. Because wound closure is a skill in which proficiency can be achieved through practice and repetition, we believe that our methods of closure can be generalized to all surgeons performing carpal tunnel releases. We used the Patient and Observer Scar Assessment Scale (POSAS), a validated scar assessment scale that measures scar quality from the patient and provider perspectives (van de Kar et al., 2005). We hypothesized that a series of interrupted, buried, deep dermal Monocryl sutures would result in equal POSAS scores compared with nylon wound closure.

## Methods

### Enrollment

**Figure 1.**
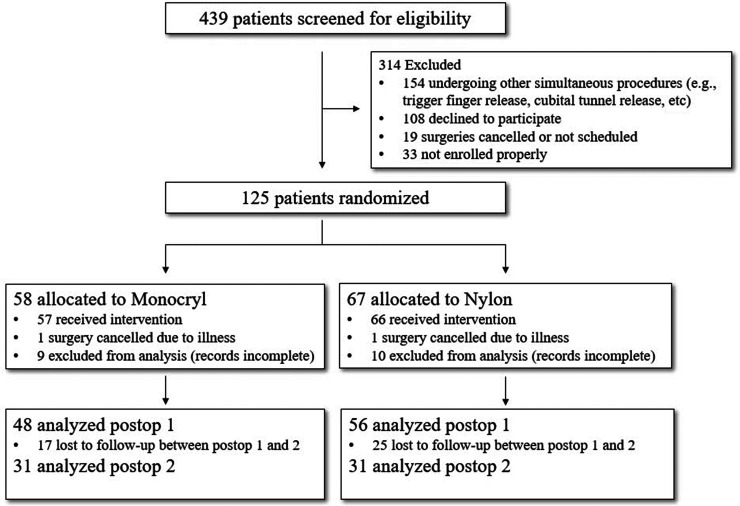
CONSORT flow chart reporting the number of patients through the enrollment, intervention, allocation, follow-up and data analysis phases of the trial.

Institutional review board approval was obtained prior to initiation of the study. Four hundred and thirty-nine patients, 18 years of age and older, undergoing carpal tunnel release by three certified hand surgeons at a single academic institution were screened for eligibility between May 2019 and October 2021. Participants were enrolled at either their preoperative clinic visit or on the day of surgery. Adults unable to provide informed consent, paediatric or adolescent patients, pregnant women and prisoners were excluded from this study. Patients undergoing simultaneous procedures on the ipsilateral extremity, such as trigger finger or cubital tunnel release, and patients with a documented allergy to adhesives or tape were also excluded. One hundred and twenty-five participants were randomized to either the subcuticular Monocryl or traditional nylon group using a digital randomizer application (Figure 1).

### Technique

Surgeries were performed by three experienced or highly-experienced fellowship-trained hand surgeons (RMS, COB, RHA) in practice for a minimum of 10 years (Tang and Giddins, 2016). Following an open carpal tunnel decompression, patients received their assigned wound closure in standardized fashion. For the Monocryl group, the wound was closed with 4-0 Monocryl in interrupted, buried, deep dermal fashion and then covered with a 2.5 cm Steri-Strip (3M, Maplewood, MN, USA) placed longitudinally over the incision. For the nylon group, the wound was closed with 4-0 nylon in interrupted, horizontal mattress fashion and then covered with Adaptic (3M, Maplewood, MN, USA). All patients received the same soft dressing postoperatively and were instructed to leave the dressing on until their first postoperative visit.

### Data collection

Patients returned for two postoperative follow-up appointments, at 10–12 days and 6 weeks after surgery. Nylon sutures were removed at the first postoperative visit. At each visit, the incisions were evaluated by trained research personnel. The treating surgeon did not perform these evaluations to ensure interobserver reliability and eliminate bias. Both the patients and research personnel evaluated the incisions via direct visualization and palpation, after which they completed POSAS evaluation. Patients assessed their scars on the criteria of pain, itchiness, colour, stiffness, thickness and irregularity, while observers rated the vascularity, pigmentation, thickness, relief, pliability and surface area of the scars. Both were also asked to provide their overall opinion of the scar. Each item was rated on a 10-point scale, with the lowest score ‘1’ corresponding to normal skin (i.e. normal pigmentation, no itching) and the highest score ‘10’ corresponding to the largest difference from normal skin (i.e. the worst imaginable scar or sensation). The scores are summed separately, with lower scores indicating closer resemblance to normal skin.

### Statistical analysis

Based on previously published data (Fleisher et al., 2019), we performed an a priori power analysis to determine the number of patients needed to adequately power our study. If we expect to see an effect size difference of 1 between absorbable versus non-absorbable sutures on the Observer Scar Assessment Scale, we determined that 141 patients would need to be enrolled in each group to have an 80% chance to detect such a difference (β = 0.8). Similarly, for the Patient Scar Assessment Scale, we determined that 63 patients would be needed in each group to have an 80% chance to detect such a difference (β = 0.8). A difference in total score of at least 1 is expected if there is a difference between groups. Results were compiled into a research database, and the de-identified data was then analysed for differences between the Monocryl and nylon methods of wound closure. For both patient and observer respondents, the data consist of six descriptive scales (scored 0–10), a sum of the six scales (0–60) and an overall opinion scale (0–10). Comparing these scales by suture (nylon versus Monocryl), timepoint (2 and 6 weeks) and respondent (patient and observer) resulted in a total of 64 groups being compared in 32 two-sample tests. The Shapiro–Wilk test of normality found that 46 of the 64 were not normally distributed. We chose accordingly to use tests that do not assume normality: the two-sample *t*-test assuming unequal variances and the nonparametric Wilcoxon rank sum test. Furthermore, prior work has demonstrated that POSAS data are non-normal (Fleisher et al., 2019). Linear regressions were performed to predict the effect of timepoint and suture type on overall opinion and total scores. Statistical significance was set at *p* < 0.05.

## Results

A total of 104 patients completed the first postoperative visit at 2 weeks, and 62 patients completed the second postoperative visit at 6 weeks. At 2 weeks, 48 patients in the Monocryl group and 56 patients in the nylon group successfully completed the questionnaires. At 6 weeks, 31 patients in the Monocryl group and 31 patients in the nylon group successfully completed the questionnaires (Figure 1). There were no surgical site complications in either group.

Average patient assessments of their scars are reported in Table 1. At 2 weeks, patients reported a statistically significant difference in thickness and irregularity between Monocryl and nylon. There was no difference in pain, itchiness, colour or stiffness between Monocryl and nylon at 2 weeks. Overall, patients receiving Monocryl had significantly better total score and opinion of their scars compared with nylon. There were no statistically significant differences in any patient assessment category between Monocryl and nylon at 6 weeks.

**Table 1.** Mean Patient Scar Assessment Scale at 2-week and 6-week postoperative visits.

	2 weeks postoperative visit			6 weeks preoperative visit		
Category	Monocryl	Nylon	*p*-value	Monocryl	Nylon	*p*-value
Pain	3.46	3.29	0.98	3.58	3.42	0.99
Itching	2.73	3.21	0.17	3.29	3.06	0.91
Colour	3.61	4.38	0.14	4.16	3.81	0.42
Stiffness	4.52	5.45	0.13	4.90	3.97	0.19
Thickness	3.87	5.41	**0.01**	4.39	3.58	0.23
Irregularity	3.66	5.34	**<0.01**	3.32	3.48	0.59
Total score	21.46	27.07	**0.02**	23.65	21.32	0.24
Overall opinion	3.55	5.09	**<0.01**	3.65	3.45	0.82

Significant *p*-value shown in bold.

Average observer assessments of the scars are reported in Table 2. At 2 weeks, observers rated scars in the Monocryl group more favourably in every category compared with scars in the nylon group, all of which reached statistical significance. By 6 weeks, observers did not report a statistically significant difference in any category between Monocryl and nylon.

**Table 2.** Mean Observer Scar Assessment Scale at 2-week and 6-week postoperative visits.

	2 weeks			6 weeks		
Category	Monocryl	Nylon	*p*-value	Monocryl	Nylon	*p*-value
Vascularity	2.52	3.08	**0.01**	2.97	3.03	0.70
Pigmentation	2.40	2.96	**0.01**	2.52	2.48	0.91
Thickness	2.74	4.00	**<0.01**	3.28	3.03	0.80
Relief	2.92	3.55	**0.03**	2.55	2.55	0.58
Pliability	3.00	3.89	**<0.01**	3.41	3.16	0.90
Surface area	2.36	3.56	**<0.01**	2.31	2.81	0.11
Total score	16.04	21.00	**<0.01**	17.03	17.06	0.56
Overall opinion	2.68	3.62	**<0.01**	2.69	2.77	0.63

Significant *p*-value shown in bold.

**Figure 2.**
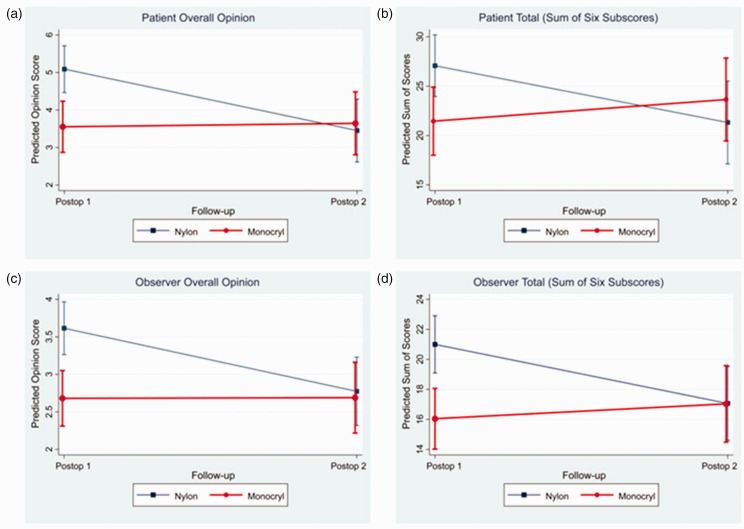
Linear regression models depicting the effect of timepoint and suture type on overall opinion and total scores. While patients and observers rated the appearance of scars closed with Monocryl similarly between 2 and 6 weeks, they noted significant improvement in the appearance of scars closed with nylon between these timepoints. No differences were noted between Monocryl and nylon at 6 weeks. Confidence intervals are shown at 95%.

Changes in patient and observer scar assessments between 2 and 6 weeks are reported for each suture type in Table 3 and Figure 2. Within the Monocryl group, there was no statistically significant change in any category rated by patients between 2 and 6 weeks. Observers noted a very slight increase in vascularity of the scars between 2 and 6 weeks. However, within the nylon group, there was a statistically significant improvement in patient ratings between the first and second postoperative visits with regards to stiffness, thickness, irregularity, total score and overall opinion. Also in the nylon group, there was a statistically significant improvement in observer ratings with regards to thickness, relief, pliability, surface area, total score and overall opinion. This demonstrates that patients and observers rated the appearance of scars closed with Monocryl very similarly at 2 and 6 weeks but noted a significant improvement between 2 and 6 weeks in the appearance of scars closed with nylon.

**Table 3.** Changes in (a) patient and (b) observer ratings for each suture type from 2 weeks to 6 weeks.

(a) Comparison of Patient Scar Assessment for each suture type from 2 weeks to 6 weeks						
	Monocryl			Nylon		
Category	2 weeks	6 weeks	*p*-value	2 weeks	6 weeks	*p*-value
Pain	3.46	3.58	0.79	3.29	3.42	0.77
Itching	2.73	3.29	0.38	3.21	3.06	0.79
Colour	3.61	4.16	0.11	4.38	3.81	0.43
Stiffness	4.52	4.90	0.47	5.45	3.97	**0.04**
Thickness	3.87	4.39	0.31	5.41	3.58	**0.01**
Irregularity	3.66	3.32	0.65	5.34	3.48	**<0.01**
Total score	21.46	23.65	0.24	27.07	21.32	**0.04**
Overall opinion	3.55	3.65	0.83	5.09	3.45	**0.01**
(b) Comparison of Observer Scar Assessment for each suture type from 2 weeks to 6 weeks.						
	Monocryl			Nylon		
Category	2 weeks	6 weeks	*p*-value	2 weeks	6 weeks	*p*-value
Vascularity	2.52	2.97	**0.05**	3.08	3.03	0.87
Pigmentation	2.40	2.52	0.42	2.96	2.48	0.11
Thickness	2.74	3.28	0.43	4.00	3.03	**<0.01**
Relief	2.92	2.55	0.23	3.55	2.55	**<0.01**
Pliability	3.00	3.41	0.33	3.89	3.16	**0.01**
Surface area	2.36	2.31	0.90	3.56	2.81	**0.01**
Total score	16.04	17.03	0.56	21.00	17.06	**<0.01**
Overall opinion	2.68	2.69	0.57	3.62	2.77	**<0.01**

Significant *p*-value shown in bold.

## Discussion

Although carpal tunnel release is the most performed elective procedure in the hand, debate remains regarding the optimal suture type and method for wound closure. There is emerging evidence that suture material and closure technique can influence the ultimate outcome (Erel et al., 2001; Fleisher et al., 2019). A recent meta-analysis of five prospective randomized controlled trials comparing absorbable and non-absorbable sutures could not conclude whether absorbable sutures conferred better, worse or equivalent outcomes compared with non-absorbable sutures due to very low quality of evidence. Furthermore, scar satisfaction was reported in just one trial (Wade et al., 2018). Our study provided evidence that patients prefer absorbable sutures in the early postoperative period, during which their scars are healing, which can inform a surgeon’s decision on how to close a carpal tunnel incision. In our experience, patient anxiety was highest at initial wound review during their first postoperative appointment. Previous studies have suggested that an uncomplicated initial recovery at 2 weeks portends a non-problematic first 6 months of recovery following carpal tunnel release (Ryan et al., 2022). Thus, optimizing patient perception early in the postoperative period while they are paying most attention to their incisions can have implications for their final outcome and recovery.

Several studies have pointed to the advantages conferred by using absorbable sutures for closure of carpal tunnel incisions. Two groups found that patients who received absorbable sutures had significantly less pain in the immediate postoperative period compared with patients who received non-absorbable sutures (Erel et al., 2001; Hansen et al., 2009). Absorbable sutures also eliminate the need for suture removal and the associated patient anxiety and discomfort (Dosani et al., 2013; Selvadurai et al., 1997). In addition, eliminating a repeat visit for suture removal benefits the healthcare system by reducing clinical workload, resource utilization and direct and indirect costs (Wade et al., 2018). Furthermore, use of absorbable sutures makes virtual visits a possibility as patients are not required to return simply for suture removal, a practice that stemmed from the COVID-19 pandemic. A recent discussion on an American Society for Surgery of the Hand (ASSH) all-member group listserv revealed that surgeons are increasingly using phone photographs sent by patients after carpal tunnel release closed with absorbable sutures and bypassing an in-person postoperative visit unless there was a problem (ASSH, 2022).

Prior economic analyses have suggested that use of absorbable sutures offers a significant opportunity for cost reduction. A Cochrane report determined that if all surgeons in the United Kingdom used non-absorbable sutures after carpal tunnel release, then the cost to the healthcare system for a nursing visit to remove sutures would be over 3 million British pounds (GBP) per year (Wade et al., 2018). This does not account for indirect costs to patients, such as time off work or travel. Regarding direct costs, a previous study suggested that the nylon suture itself was cheaper (5 GBP versus 1.64 GBP), thereby making it the more cost-effective option (Dosani et al., 2013). However, at our academic surgery centre, the cost of one 4-0 nylon suture is 3.23 United States Dollars (USD) compared with 4.64 USD for one 4-0 Monocryl. This is a marginal difference of 1.41 USD per wound, which extrapolated to a year, remains far less costly than in-person return visits for suture removal. Our study confirms that Monocryl has equal or better cosmetic outcomes than nylon, and it is potentially the more cost-effective option by making virtual visits a possibility. Patient opinions regarding telemedicine in the hand and upper extremity clinic have been quite favourable (Benavent et al., 2022). The option for a virtual postoperative visit without the need for suture removal may thus be preferrable and provide substantial cost and time savings for patients, providers and healthcare institutions.

Prior studies have highlighted potential drawbacks of absorbable sutures, namely increased residual wound inflammation and persistent scar tenderness (Kirpensteijn et al., 1997; Menovsky et al., 2004; Niessen et al., 1997). However, these early studies used Vicryl or Vicryl Rapide (polyglactin 910, Ethicon, Inc., Raritan, NJ, USA) as opposed to Monocryl. Monocryl has been associated with reduced tissue reaction and inflammation, and has been shown to produce significantly smaller and less hypertrophic scars compared with Vicryl (Niessen et al., 1997). In another recent study, incisions closed with Monocryl in the same fashion as our study (a series of interrupted, deep dermal, 4-0 sutures) were significantly less likely to develop dehiscence, infection or lead to additional wound-related encounters compared with incisions closed with 4-0 nylon or 4-0 chromic gut in mattress fashion (Rochlin et al., 2019).

**Figure 3.**
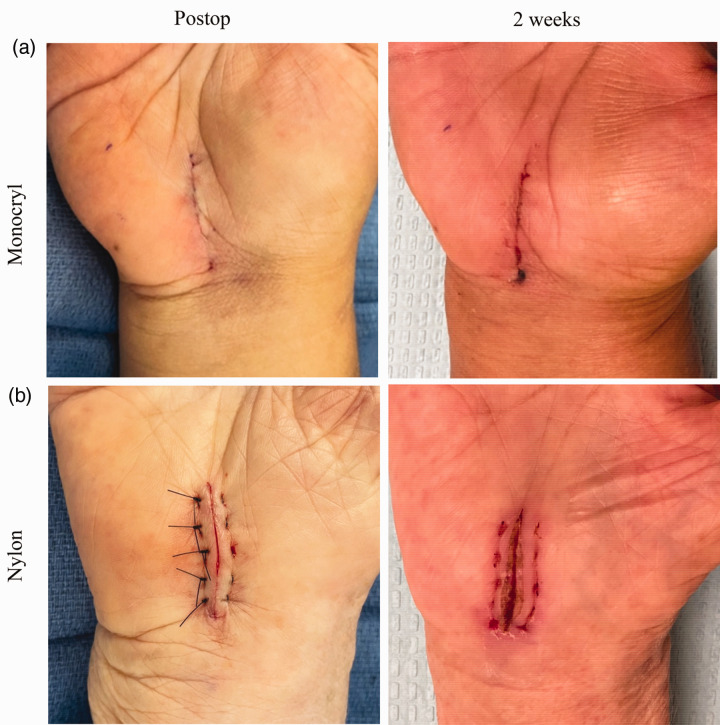
Scar appearance following Monocryl (a) and nylon (b) wound closures immediately postoperative and at 2 weeks.

We found no differences in patient or observer assessment of any category between the Monocryl or nylon groups at 6 weeks, although our study was underpowered to reveal if there actually would be a difference in observer ratings at this timepoint. No patient in our study developed a delayed adverse reaction to Monocryl. No patients developed any surgical site complications. This suggests that delayed tissue reaction or inflammation is not a major concern at 6 weeks following Monocryl use and that absorbable and non-absorbable sutures produce comparable scars in the long-term. Furthermore, our findings challenge the classic teaching that non-absorbable sutures cause less inflammation and therefore result in a better scar. Figure 3 shows the typical appearance of two scars immediately following closure in the operating room and at the 2-week follow-up. Based on our observations and the objective results of our study, it is our opinion that Monocryl potentially produces a better cosmetic result compared with nylon in the early postoperative period when patients are most aware of their scars.

Our study has limitations. Based on our a priori power analysis, our study was underpowered to detect a difference in patient and observer scar assessment scales between Monocryl and nylon at 6 weeks. However, it is our experience and impression while conducting this study that the majority of patients in both groups were fully healed by 6 weeks with only minimally noticeable difference in their scars. Indeed, many patients questioned the need for the 6-week postoperative appointment, and only 62 of the 104 patients returned to complete the 6-week study visit. We do not believe that those patients lost to follow-up would bias our results, as they likely had satisfactory healing of their scars, which would reinforce the findings that no major differences exist between Monocryl and nylon closures at 6 weeks and that similar long-term outcomes can be expected regardless of suture type or closure technique. Recruiting an additional 83 patients to power the study to detect a difference in observer scar ratings at 6 weeks did not seem like a practical endeavour. Importantly, we did find statistically significant differences between Monocryl and nylon at 2 weeks. When statistical tests are significant, it is always of sufficient power, and thus we can reject our null hypothesis that Monocryl and nylon would produce equal POSAS scores at 2 weeks. Another limitation is that we were unable to perform a multivariate analysis of other surgical variables that might have influenced scar appearance (e.g. time to suture removal, tightness of closure). A final limitation is that 6 weeks may be considered a relatively short follow-up period. However, we anticipated that because most wounds would be fully healed at this stage, it would be challenging to have patients return several months later beyond the normal follow-up period for a carpal tunnel release, which did turn out to be the case. Furthermore, at 6 weeks wounds are nearing the end of the proliferative healing phase before remodelling, and thus any differences between groups would be best highlighted in the period leading up to this timepoint (Theopold et al., 2012). Our results corroborate the findings of prior studies that carpal tunnel incisions heal reliably with similar outcomes in the long-term, and thus we do not believe that longer follow-up would add substantial findings when evaluating the effects of suture type or closure technique on wound healing.

## Supplemental Material

sj-zip-1-jhs-10.1177_17531934231178383 - Supplemental material for Prospective randomized controlled trial comparing the effect of Monocryl versus nylon sutures on patient- and observer-assessed outcomes following carpal tunnel surgery Click here for additional data file. Supplemental material, sj-zip-1-jhs-10.1177_17531934231178383 for Prospective randomized controlled trial comparing the effect of Monocryl versus nylon sutures on patient- and observer-assessed outcomes following carpal tunnel surgery by Edward Wu, Robert Allen, Christopher Bayne and Robert Szabo in Journal of Hand Surgery (European Volume)
