# Supplementary material for: Prospective randomized controlled trial comparing the effect of Monocryl versus nylon sutures on patient- and observer-assessed outcomes following carpal tunnel surgery
Source: J Hand Surg Eur Vol. 2023 Jun 9;48(10):1014–21. doi: 10.1177/17531934231178383 (PMC10616990; doi:10.1177/17531934231178383)
Supplement: sj-zip-1-jhs-10.1177_17531934231178383 - Supplemental material for Prospective randomized controlled trial comparing the effect of Monocryl versus nylon sutures on patient- and observer-assessed outcomes following carpal tunnel surgery [file sj-zip-1-jhs-10.1177_17531934231178383.zip › Supplementary Materials/results_regressions_2023-02-21.docx]

*** Carpal Tunnel Closure Data**

*** February 2023**

*** Dr. Ed Wu**

*** 21 February 2023**

* Data file: data_2023-02-10.dta

**Significance**

P < 0.01** Strongly significant

P < 0.05* Significant

P < 0.1 Trend

P < 0.2 Weak trend

MULTIPLE LINEAR REGRESSIONS OF OUTCOME ON F/U AND SUTURE TYPE

F/U : Postop1 = 2 weeks, Postop2 = 6 weeks

Suture : Nylon, Monocryl

Except for the first margins report, I’ve left out main effects to save space. Main effects are average scores of one factor across all levels of the other factor. We want the more detailed (interaction) breakdown.

The graphics here can be made prettier in PowerPoint. Let me know which you want to use and any changes you’d like to see.

- Paul

************************************************************************************

PATIENT OVERALL OPINION

regress opin_pt wk##suture, baselevels

Source | SS df MS Number of obs = 165

-------------+--------------------------------- F(3, 161) = 5.25

Model | 88.1824865 3 29.3941622 Prob > F = 0.0017**

Residual | 900.944786 161 5.59593035 R-squared = 0.0892

-------------+--------------------------------- Adj R-squared = 0.0722 7% explained var.

Total | 989.127273 164 6.03126386 Root MSE = 2.3656

------------------------------------------------------------------------------------

opin_pt | Coefficient Std. err. t P>|t| [95% conf. interval]

-------------------+---------------------------------------------------------------

wks |

Postop 1 | 0 (base)

Postop 2 | -1.637673 .529567 -3.09 0.002** -2.683466 -.5918796

|

suture |

Nylon | 0 (base)

Monocryl | -1.536094 .4679633 -3.28 0.001** -2.460232 -.6119565

|

wks#suture |

Postop 2#Monocryl | 1.729643 .7615888 2.27 0.024* .225651 3.233634

|

_cons | 5.089286 .3161128 16.10 0.000 4.465024 5.713548

------------------------------------------------------------------------------------

The interaction is significant.

Table of predicted Patient Overall Opinion by F/U and Suture Type:

Predictive margins Number of obs = 165

Model VCE: OLS

Expression: Linear prediction, predict(). Main effects and interactions.

------------------------------------------------------------------------------------

| Delta-method

| Margin std. err. t P>|t| [95% conf. interval]

-------------------+---------------------------------------------------------------

wks |

Postop 1 | 4.363132 .2332133 18.71 0.000 3.902581 4.823684

Postop 2 | 3.543109 .3008745 11.78 0.000 2.948939 4.137278

|

suture |

Nylon | 4.473918 .2538247 17.63 0.000 3.972663 4.975173

Monocryl | 3.58775 .268111 13.38 0.000 3.058282 4.117218

|

wks#suture |

Postop 1#Nylon | 5.089286 .3161128 16.10 0.000 4.465024 5.713548

Postop 1#Monocryl | 3.553191 .3450541 10.30 0.000 2.871776 4.234607

Postop 2#Nylon | 3.451613 .4248693 8.12 0.000 2.612578 4.290648

Postop 2#Monocryl | 3.645161 .4248693 8.58 0.000 2.806126 4.484197

------------------------------------------------------------------------------------

Table of predicted Patient Overall Opinion by F/U and Suture Type:

Predictive margins Number of obs = 165

**LEAVING OUT THE MAIN EFFECTS (all remaining breakdowns will leave out main effects):**

------------------------------------------------------------------------------------

| Delta-method

| Margin std. err. t P>|t| [95% conf. interval]

-------------------+---------------------------------------------------------------

wks#suture |

Postop 1#Nylon | 5.089286 .3161128 16.10 0.000 4.465024 5.713548

Postop 1#Monocryl | 3.553191 .3450541 10.30 0.000 2.871776 4.234607

Postop 2#Nylon | 3.451613 .4248693 8.12 0.000 2.612578 4.290648

Postop 2#Monocryl | 3.645161 .4248693 8.58 0.000 2.806126 4.484197

------------------------------------------------------------------------------------

**P>|t| tests the margin's difference from zero, which doesn't interest us.**

************************************************************************************

PATIENT TOTAL SCORE (SUM OF SIX SUBSCORES)

regress pt_sum wk##suture, baselevels

Source | SS df MS Number of obs = 164

-------------+--------------------------------- F(3, 160) = 2.48

Model | 1042.72731 3 347.575771 Prob > F = 0.0630 Fairly strong trend.

Residual | 22418.9983 160 140.118739 R-squared = 0.0444

-------------+--------------------------------- Adj R-squared = 0.0265 3% explained var.

Total | 23461.7256 163 143.936967 Root MSE = 11.837

------------------------------------------------------------------------------------

pt_sum | Coefficient Std. err. t P>|t| [95% conf. interval]

-------------------+---------------------------------------------------------------

wks |

Postop 1 | 0 (base)

Postop 2 | -5.748848 2.649921 -2.17 0.032* -10.98218 -.5155154

|

suture |

Nylon | 0 (base)

Monocryl | -5.614907 2.355457 -2.38 0.018* -10.2667 -.9631102

|

wks#suture |

Postop 2#Monocryl | 7.937487 3.819437 2.08 0.039* .3944755 15.4805

|

_cons | 27.07143 1.581809 17.11 0.000 23.94751 30.19535

------------------------------------------------------------------------------------

The interaction is significant.

Table of predicted Patient Total Score by F/U and Suture Type:

Predictive margins Number of obs = 164

------------------------------------------------------------------------------------

| Delta-method

| Margin std. err. t P>|t| [95% conf. interval]

-------------------+---------------------------------------------------------------

wks#suture |

Postop 1#Nylon | 27.07143 1.581809 17.11 0.000 23.94751 30.19535

Postop 1#Monocryl | 21.45652 1.745296 12.29 0.000 18.00973 24.90331

Postop 2#Nylon | 21.32258 2.12602 10.03 0.000 17.1239 25.52126

Postop 2#Monocryl | 23.64516 2.12602 11.12 0.000 19.44648 27.84384

------------------------------------------------------------------------------------

************************************************************************************

OBSERVER OVERALL OPINION

regress opin_obs wk##suture, baselevels

Source | SS df MS Number of obs = 159

-------------+--------------------------------- F(3, 155) = 5.82

Model | 28.847001 3 9.61566701 Prob > F = 0.0009**

Residual | 256.14671 155 1.65255942 R-squared = 0.1012

-------------+--------------------------------- Adj R-squared = 0.0838 8% explained var.

Total | 284.993711 158 1.80375766 Root MSE = 1.2855

------------------------------------------------------------------------------------

opin_obs | Coefficient Std. err. t P>|t| [95% conf. interval]

-------------------+---------------------------------------------------------------

wks |

Postop 1 | 0 (base)

Postop 2 | -.8411911 .2916991 -2.88 0.004** -1.41741 -.2649724

|

suture |

Nylon | 0 (base)

Monocryl | -.9345336 .2587293 -3.61 0.000** -1.445624 -.4234431

|

wks#suture |

Postop 2#Monocryl | .8499952 .4209917 2.02 0.045* .0183736 1.681617

|

_cons | 3.615385 .1782694 20.28 0.000 3.263233 3.967536

------------------------------------------------------------------------------------

The interaction is significant.

Table of predicted Observer Overall Opinion by F/U and Suture Type:

Predictive margins Number of obs = 159

------------------------------------------------------------------------------------

| Delta-method

| Margin std. err. t P>|t| [95% conf. interval]

-------------------+---------------------------------------------------------------

wks#suture |

Postop 1#Nylon | 3.615385 .1782694 20.28 0.000 3.263233 3.967536

Postop 1#Monocryl | 2.680851 .1875122 14.30 0.000 2.310442 3.05126

Postop 2#Nylon | 2.774194 .2308861 12.02 0.000 2.318104 3.230283

Postop 2#Monocryl | 2.689655 .2387149 11.27 0.000 2.218101 3.16121

------------------------------------------------------------------------------------

************************************************************************************

OBSERVER TOTAL SCORE (SUM OF SIX SUBSCORES)

regress obs_sum wk##suture, baselevels

Source | SS df MS Number of obs = 158

-------------+--------------------------------- F(3, 154) = 4.81

Model | 697.737813 3 232.579271 Prob > F = 0.0031**

Residual | 7438.74953 154 48.3035684 R-squared = 0.0858

-------------+--------------------------------- Adj R-squared = 0.0679 7% explained var.

Total | 8136.48734 157 51.8247601 Root MSE = 6.9501

------------------------------------------------------------------------------------

obs_sum | Coefficient Std. err. t P>|t| [95% conf. interval]

-------------------+---------------------------------------------------------------

wks |

Postop 1 | 0 (base)

Postop 2 | -3.935484 1.577052 -2.50 0.014* -7.050932 -.8200356

|

suture |

Nylon | 0 (base)

Monocryl | -4.956522 1.406767 -3.52 0.001** -7.735572 -2.177471

|

wks#suture |

Postop 2#Monocryl | 4.926488 2.280967 2.16 0.032* .4204645 9.432512

|

_cons | 21 .9638022 21.79 0.000 19.09602 22.90398

------------------------------------------------------------------------------------

The interaction is significant.

Table of predicted Observer Total Score by F/U and Suture Type:

Predictive margins Number of obs = 158

------------------------------------------------------------------------------------

| Delta-method

| Margin std. err. t P>|t| [95% conf. interval]

-------------------+---------------------------------------------------------------

wks#suture |

Postop 1#Nylon | 21 .9638022 21.79 0.000 19.09602 22.90398

Postop 1#Monocryl | 16.04348 1.024733 15.66 0.000 14.01913 18.06783

Postop 2#Nylon | 17.06452 1.248271 13.67 0.000 14.59857 19.53046

Postop 2#Monocryl | 17.03448 1.290597 13.20 0.000 14.48492 19.58404

------------------------------------------------------------------------------------
