# Supplementary material for: Prospective randomized controlled trial comparing the effect of Monocryl versus nylon sutures on patient- and observer-assessed outcomes following carpal tunnel surgery
Source: J Hand Surg Eur Vol. 2023 Jun 9;48(10):1014–21. doi: 10.1177/17531934231178383 (PMC10616990; doi:10.1177/17531934231178383)
Supplement: sj-zip-1-jhs-10.1177_17531934231178383 - Supplemental material for Prospective randomized controlled trial comparing the effect of Monocryl versus nylon sutures on patient- and observer-assessed outcomes following carpal tunnel surgery [file sj-zip-1-jhs-10.1177_17531934231178383.zip › Supplementary Materials/results_2-sample_po1_vs_po2_2023-02-27_corrected.pdf]

* Carpal Tunnel Closure Data
* February 2023
* Dr. Ed Wu
* 27 February 2023
* Data file: data_2023-02-10.dta

* 2-Sample Tests: Postop 1 vs. Postop 2 for Nylon and Monocryl within each POSAS score category.
   1) 2-sample t tests of Po 1 vs. Po 2 with ***unequal variance*** option
      This is a correction of the 10 February run which mistakenly assumed equal variances.
   2) nonparametric rank sum tests of Po 1 vs. Po 2

Notes:
2-sample tests across follow-up (Postop 1 vs. Postop 2) do not take patient-specific error into account, as paired tests would, but they maximize the number of included patients in Postop 1.

Significance
P < 0.01   Strongly significant**
P < 0.05*   Significant
P < 0.1   Trend
P < 0.2   Weak trend

```
******************************************************************************
* Patient responses by Suture and F/U

* ============================================================================ Pt Pain
* Patient:  Pain
by suture, sort : ttest pain, by(wks) unequal welch

------------------------------------------------------------------------------
-> suture = Nylon

Two-sample t test with unequal variances
------------------------------------------------------------------------------
   Group |     Obs        Mean    Std. err.   Std. dev.   [95% conf. interval]
---------+--------------------------------------------------------------------
Postop 1 |      56    3.285714    .2656947    1.988277     2.75325    3.818178
Postop 2 |      31    3.419355    .3817734    2.125625    2.639669     4.19904
---------+--------------------------------------------------------------------
Combined |      87    3.333333    .2173119     2.02695    2.901332    3.765335
---------+--------------------------------------------------------------------
    diff |           -.1336406    .4651286               -1.063941      .79666
------------------------------------------------------------------------------
    diff = mean(Postop 1) - mean(Postop 2)                        t =  -0.2873
H0: diff = 0                             Welch's degrees of freedom =     60.3

    Ha: diff < 0                 Ha: diff != 0                 Ha: diff > 0
 Pr(T < t) = 0.3874         Pr(|T| > |t|) = 0.7749          Pr(T > t) = 0.6126

Two-sample Wilcoxon rank-sum (Mann-Whitney) test
H0: pain(wks==Postop 1) = pain(wks==Postop 2)
         z = -0.289
Prob > |z| = 0.7729

------------------------------------------------------------------------------
-> suture = Monocryl

Two-sample t test with unequal variances
------------------------------------------------------------------------------
   Group |     Obs        Mean    Std. err.   Std. dev.   [95% conf. interval]
---------+--------------------------------------------------------------------
Postop 1 |      48    3.458333    .3407313    2.360656     2.77287    4.143796
Postop 2 |      31    3.580645    .4294888    2.391292    2.703512    4.457778
---------+--------------------------------------------------------------------
Combined |      79    3.506329    .2653123    2.358147    2.978133    4.034525
---------+--------------------------------------------------------------------
    diff |           -.1223118    .5482321                -1.21705    .9724261
------------------------------------------------------------------------------
    diff = mean(Postop 1) - mean(Postop 2)                        t =  -0.2231
H0: diff = 0                             Welch's degrees of freedom =  65.4961

    Ha: diff < 0                 Ha: diff != 0                 Ha: diff > 0
 Pr(T < t) = 0.4121         Pr(|T| > |t|) = 0.8242          Pr(T > t) = 0.5879

Two-sample Wilcoxon rank-sum (Mann-Whitney) test
H0: pain(wks==Postop 1) = pain(wks==Postop 2)
         z = -0.270
Prob > |z| = 0.7869
```

```
* ============================================================================= Pt Itchiness
* Patient:  Scar Itchiness
by suture, sort : ttest itch, by(wks) unequal welch

-------------------------------------------------------------------------------
-> suture = Nylon

Two-sample t test with unequal variances
------------------------------------------------------------------------------
   Group |     Obs        Mean    Std. err.   Std. dev.   [95% conf. interval]
---------+--------------------------------------------------------------------
Postop 1 |      56    3.214286    .2935611     2.19681    2.625976    3.802595
Postop 2 |      31    3.064516    .3850303    2.143758    2.278179    3.850853
---------+--------------------------------------------------------------------
Combined |      87     3.16092    .2322982    2.166733    2.699126    3.622713
---------+--------------------------------------------------------------------
    diff |            .1497696    .4841761               -.8171244    1.116664
------------------------------------------------------------------------------
    diff = mean(Postop 1) - mean(Postop 2)                        t =   0.3093
H0: diff = 0                                 Welch's degrees of freedom =  65.2578

    Ha: diff < 0                 Ha: diff != 0                 Ha: diff > 0
 Pr(T < t) = 0.6210         Pr(|T| > |t|) = 0.7581          Pr(T > t) = 0.3790

Two-sample Wilcoxon rank-sum (Mann-Whitney) test
H0: itch(wks==Postop 1) = itch(wks==Postop 2)
         z =  0.267
Prob > |z| = 0.7895

-------------------------------------------------------------------------------
-> suture = Monocryl

Two-sample t test with unequal variances
------------------------------------------------------------------------------
   Group |     Obs        Mean    Std. err.   Std. dev.   [95% conf. interval]
---------+--------------------------------------------------------------------
Postop 1 |      48    2.729167    .3189349    2.209646    2.087552    3.370781
Postop 2 |      31    3.290323    .4733626    2.635571    2.323587    4.257058
---------+--------------------------------------------------------------------
Combined |      79    2.949367    .2683685    2.385312    2.415086    3.483648
---------+--------------------------------------------------------------------
    diff |           -.5611559    .5707816               -1.703858    .5815465
------------------------------------------------------------------------------
    diff = mean(Postop 1) - mean(Postop 2)                        t =  -0.9831
H0: diff = 0                                 Welch's degrees of freedom =  57.6236

    Ha: diff < 0                 Ha: diff != 0                 Ha: diff > 0
 Pr(T < t) = 0.1648         Pr(|T| > |t|) = 0.3297          Pr(T > t) = 0.8352

Two-sample Wilcoxon rank-sum (Mann-Whitney) test
H0: itch(wks==Postop 1) = itch(wks==Postop 2)
         z = -0.887
Prob > |z| = 0.3751
```

```
* ============================================================================ Pt Color
* Patient:  Scar Color
by suture, sort : ttest color, by(wks) unequal welch

-------------------------------------------------------------------------------
-> suture = Nylon

Two-sample t test with unequal variances
------------------------------------------------------------------------------
   Group |     Obs        Mean    Std. err.   Std. dev.   [95% conf. interval]
---------+--------------------------------------------------------------------
Postop 1 |      56       4.375    .3911044    2.926757    3.591209    5.158791
Postop 2 |      31    3.806452    .4693151    2.613036    2.847982    4.764921
---------+--------------------------------------------------------------------
Combined |      87    4.172414    .3020056    2.816921    3.572046    4.772781
---------+--------------------------------------------------------------------
    diff |            .5685484    .6109168               -.6497945    1.786891
------------------------------------------------------------------------------
    diff = mean(Postop 1) - mean(Postop 2)                        t =   0.9306
H0: diff = 0                                 Welch's degrees of freedom =  70.3029

    Ha: diff < 0                 Ha: diff != 0                 Ha: diff > 0
 Pr(T < t) = 0.8224         Pr(|T| > |t|) = 0.3552          Pr(T > t) = 0.1776

Two-sample Wilcoxon rank-sum (Mann-Whitney) test
H0: color(wks==Postop 1) = color(wks==Postop 2)
         z =  0.797
Prob > |z| = 0.4254

-------------------------------------------------------------------------------
-> suture = Monocryl

Two-sample t test with unequal variances
------------------------------------------------------------------------------
   Group |     Obs        Mean    Std. err.   Std. dev.   [95% conf. interval]
---------+--------------------------------------------------------------------
Postop 1 |      46    3.608696    .4379114     2.97006    2.726697    4.490695
Postop 2 |      31     4.16129    .4252685    2.367795    3.292776    5.029804
---------+--------------------------------------------------------------------
Combined |      77    3.831169    .3123142    2.740546    3.209141    4.453197
---------+--------------------------------------------------------------------
    diff |           -.5525947    .6104258               -1.768641    .6634515
------------------------------------------------------------------------------
    diff = mean(Postop 1) - mean(Postop 2)                        t =  -0.9053
H0: diff = 0                                 Welch's degrees of freedom =  74.9416

    Ha: diff < 0                 Ha: diff != 0                 Ha: diff > 0
 Pr(T < t) = 0.1841         Pr(|T| > |t|) = 0.3682          Pr(T > t) = 0.8159

Two-sample Wilcoxon rank-sum (Mann-Whitney) test
H0: color(wks==Postop 1) = color(wks==Postop 2)
         z = -1.604
Prob > |z| = 0.1086 Po 1 < Po 2 for Monocryl, but it’s a weak trend. Poor evidence.
```

```
* ============================================================================ Pt Stiffness
* Patient:  Scar Stiffness
by suture, sort : ttest stiffn, by(wks) unequal welch

------------------------------------------------------------------------------
-> suture = Nylon

Two-sample t test with unequal variances
------------------------------------------------------------------------------
   Group |     Obs        Mean    Std. err.   Std. dev.   [95% conf. interval]
---------+--------------------------------------------------------------------
Postop 1 |      56    5.446429    .4004216    2.996481    4.643966    6.248891
Postop 2 |      31    3.967742    .4029031     2.24327    3.144904     4.79058
---------+--------------------------------------------------------------------
Combined |      87     4.91954    .3033355    2.829325    4.316529    5.522551
---------+--------------------------------------------------------------------
    diff |            1.478687     .568039                .3481856    2.609188
------------------------------------------------------------------------------
    diff = mean(Postop 1) - mean(Postop 2)                        t =   2.6031
H0: diff = 0                                 Welch's degrees of freedom =  79.6909

    Ha: diff < 0                 Ha: diff != 0                 Ha: diff > 0
 Pr(T < t) = 0.9945         Pr(|T| > |t|) = 0.0110*         Pr(T > t) = 0.0055

Two-sample Wilcoxon rank-sum (Mann-Whitney) test
H0: stiffn(wks==Postop 1) = stiffn(wks==Postop 2)
         z =  2.070
Prob > |z| = 0.0385* Po 2 < Po 1 for Nylon

------------------------------------------------------------------------------
-> suture = Monocryl

Two-sample t test with unequal variances
------------------------------------------------------------------------------
   Group |     Obs        Mean    Std. err.   Std. dev.   [95% conf. interval]
---------+--------------------------------------------------------------------
Postop 1 |      48    4.520833    .4178949    2.895261    3.680137    5.361529
Postop 2 |      31    4.903226    .4759932    2.650218    3.931118    5.875334
---------+--------------------------------------------------------------------
Combined |      79    4.670886    .3139727    2.790651    4.045814    5.295958
---------+--------------------------------------------------------------------
    diff |           -.3823925     .633408               -1.645592    .8808074
------------------------------------------------------------------------------
    diff = mean(Postop 1) - mean(Postop 2)                        t =  -0.6037
H0: diff = 0                                 Welch's degrees of freedom =  70.2928

    Ha: diff < 0                 Ha: diff != 0                 Ha: diff > 0
 Pr(T < t) = 0.2740         Pr(|T| > |t|) = 0.5480          Pr(T > t) = 0.7260

Two-sample Wilcoxon rank-sum (Mann-Whitney) test
H0: stiffn(wks==Postop 1) = stiffn(wks==Postop 2)
         z = -0.718
Prob > |z| = 0.4726
```

```
* ============================================================================= Pt Thickness
* Patient:  Scar Thickness
by suture, sort : ttest thick_pt, by(wks) unequal welch

------------------------------------------------------------------------------
-> suture = Nylon

Two-sample t test with unequal variances
------------------------------------------------------------------------------
   Group |     Obs        Mean    Std. err.   Std. dev.   [95% conf. interval]
---------+--------------------------------------------------------------------
Postop 1 |      56    5.410714    .3954094    2.958973    4.618296    6.203132
Postop 2 |      31    3.580645    .3760982    2.094026     2.81255     4.34874
---------+--------------------------------------------------------------------
Combined |      87    4.758621    .3014558    2.811792    4.159346    5.357895
---------+--------------------------------------------------------------------
    diff |            1.830069    .5457091                .7445059    2.915632
------------------------------------------------------------------------------
    diff = mean(Postop 1) - mean(Postop 2)                        t =   3.3536
H0: diff = 0                             Welch's degrees of freedom =  82.1316

    Ha: diff < 0                 Ha: diff != 0                 Ha: diff > 0
 Pr(T < t) = 0.9994         Pr(|T| > |t|) = 0.0012**         Pr(T > t) = 0.0006

Two-sample Wilcoxon rank-sum (Mann-Whitney) test
H0: thick_pt(wks==Postop 1) = thick_pt(wks==Postop 2)
         z =  2.782
Prob > |z| = 0.0054** Po 2 < Po 1 for Nylon

------------------------------------------------------------------------------
-> suture = Monocryl

Two-sample t test with unequal variances
------------------------------------------------------------------------------
   Group |     Obs        Mean    Std. err.   Std. dev.   [95% conf. interval]
---------+--------------------------------------------------------------------
Postop 1 |      47     3.87234    .4110151    2.817778     3.04501    4.699671
Postop 2 |      31    4.387097    .4629903    2.577821    3.441544    5.332649
---------+--------------------------------------------------------------------
Combined |      78    4.076923     .307942     2.71967    3.463732    4.690114
---------+--------------------------------------------------------------------
    diff |           -.5147563     .619107               -1.749417    .7199045
------------------------------------------------------------------------------
    diff = mean(Postop 1) - mean(Postop 2)                        t =  -0.8314
H0: diff = 0                             Welch's degrees of freedom =  70.3537

    Ha: diff < 0                 Ha: diff != 0                 Ha: diff > 0
 Pr(T < t) = 0.2043         Pr(|T| > |t|) = 0.4085          Pr(T > t) = 0.7957

Two-sample Wilcoxon rank-sum (Mann-Whitney) test
H0: thick_pt(wks==Postop 1) = thick_pt(wks==Postop 2)
         z = -1.021
Prob > |z| = 0.3071
```

```
* ============================================================================ Pt Irregularity
* Patient:  Scar Irregularity
by suture, sort : ttest irreg, by(wks) unequal welch

------------------------------------------------------------------------------
-> suture = Nylon

Two-sample t test with unequal variances
------------------------------------------------------------------------------
   Group |     Obs        Mean    Std. err.   Std. dev.   [95% conf. interval]
---------+--------------------------------------------------------------------
Postop 1 |      56    5.339286     .400711    2.998647    4.536243    6.142329
Postop 2 |      31    3.483871    .4092242    2.278464    2.648124    4.319618
---------+--------------------------------------------------------------------
Combined |      87    4.678161    .3099932    2.891424    4.061915    5.294407
---------+--------------------------------------------------------------------
    diff |            1.855415    .5727423                .7153964    2.995433
------------------------------------------------------------------------------
    diff = mean(Postop 1) - mean(Postop 2)                        t =   3.2395
H0: diff = 0                                  Welch's degrees of freedom =  78.9854

    Ha: diff < 0                 Ha: diff != 0                 Ha: diff > 0
 Pr(T < t) = 0.9991         Pr(|T| > |t|) = 0.0018**         Pr(T > t) = 0.0009

Two-sample Wilcoxon rank-sum (Mann-Whitney) test
H0: irreg(wks==Postop 1) = irreg(wks==Postop 2)
         z =  2.892
Prob > |z| = 0.0038** Po 2 < Po 1 for Nylon

------------------------------------------------------------------------------
-> suture = Monocryl

Two-sample t test with unequal variances
------------------------------------------------------------------------------
   Group |     Obs        Mean    Std. err.   Std. dev.   [95% conf. interval]
---------+--------------------------------------------------------------------
Postop 1 |      47    3.659574    .4095039    2.807417    2.835286    4.483863
Postop 2 |      31    3.322581    .4310205    2.399821    2.442319    4.202842
---------+--------------------------------------------------------------------
Combined |      78    3.525641    .2991409    2.641941    2.929975    4.121307
---------+--------------------------------------------------------------------
    diff |            .3369938    .5945352               -.8478948    1.521882
------------------------------------------------------------------------------
    diff = mean(Postop 1) - mean(Postop 2)                        t =   0.5668
H0: diff = 0                                  Welch's degrees of freedom =  73.0672

    Ha: diff < 0                 Ha: diff != 0                 Ha: diff > 0
 Pr(T < t) = 0.7137         Pr(|T| > |t|) = 0.5726         Pr(T > t) = 0.2863

Two-sample Wilcoxon rank-sum (Mann-Whitney) test
H0: irreg(wks==Postop 1) = irreg(wks==Postop 2)
         z =  0.448
Prob > |z| = 0.6538
```

```
* ============================================================================== Pt Overall Opinion
* Patient:  Overall Opinion
by suture, sort : ttest opin_pt, by(wks) unequal welch

-------------------------------------------------------------------------------
-> suture = Nylon

Two-sample t test with unequal variances
------------------------------------------------------------------------------
   Group |     Obs        Mean    Std. err.   Std. dev.   [95% conf. interval]
---------+--------------------------------------------------------------------
Postop 1 |      56    5.089286    .3633152    2.718802    4.361186    5.817386
Postop 2 |      31    3.451613    .3338878    1.859009    2.769723    4.133503
---------+--------------------------------------------------------------------
Combined |      87    4.505747    .2744943    2.560312    3.960071    5.051424
---------+--------------------------------------------------------------------
    diff |            1.637673    .4934359                .6563207    2.619025
------------------------------------------------------------------------------
    diff = mean(Postop 1) - mean(Postop 2)                        t =   3.3189
H0: diff = 0                                Welch's degrees of freedom =  83.4146

    Ha: diff < 0                 Ha: diff != 0                 Ha: diff > 0
 Pr(T < t) = 0.9993         Pr(|T| > |t|) = 0.0013**          Pr(T > t) = 0.0007

Two-sample Wilcoxon rank-sum (Mann-Whitney) test
H0: opin_pt(wks==Postop 1) = opin_pt(wks==Postop 2)
         z =  2.775
Prob > |z| = 0.0055** Po 2 < Po 1 for Nylon**

-------------------------------------------------------------------------------
-> suture = Monocryl

Two-sample t test with unequal variances
------------------------------------------------------------------------------
   Group |     Obs        Mean    Std. err.   Std. dev.   [95% conf. interval]
---------+--------------------------------------------------------------------
Postop 1 |      47    3.553191    .3370555    2.310736    2.874734    4.231649
Postop 2 |      31    3.645161    .3949912    2.199218    2.838482    4.451841
---------+--------------------------------------------------------------------
Combined |      78    3.589744    .2551082    2.253055    3.081758    4.097729
---------+--------------------------------------------------------------------
    diff |           -.0919698    .5192537               -1.127958    .9440181
------------------------------------------------------------------------------
    diff = mean(Postop 1) - mean(Postop 2)                        t =  -0.1771
H0: diff = 0                                Welch's degrees of freedom =  68.6101

    Ha: diff < 0                 Ha: diff != 0                 Ha: diff > 0
 Pr(T < t) = 0.4300         Pr(|T| > |t|) = 0.8599         Pr(T > t) = 0.5700

Two-sample Wilcoxon rank-sum (Mann-Whitney) test
H0: opin_pt(wks==Postop 1) = opin_pt(wks==Postop 2)
         z = -0.218
Prob > |z| = 0.8274
```

```
* ============================================================================ Pt Total of 6
* Patient:  Total of 6 Scores
by suture, sort : ttest pt_sum, by(wks) unequal welch

-------------------------------------------------------------------------------
-> suture = Nylon

Two-sample t test with unequal variances
------------------------------------------------------------------------------
   Group |     Obs        Mean    Std. err.   Std. dev.   [95% conf. interval]
---------+--------------------------------------------------------------------
Postop 1 |      56    27.07143    1.713366    12.82165    23.63777    30.50509
Postop 2 |      31    21.32258    2.033794    11.32368    17.16902    25.47614
---------+--------------------------------------------------------------------
Combined |      87    25.02299    1.345637    12.55127    22.34795    27.69803
---------+--------------------------------------------------------------------
    diff |            5.748848    2.659312                .4462278    11.05147
------------------------------------------------------------------------------
    diff = mean(Postop 1) - mean(Postop 2)                        t =   2.1618
H0: diff = 0                                Welch's degrees of freedom =  70.9201

    Ha: diff < 0                 Ha: diff != 0                 Ha: diff > 0
 Pr(T < t) = 0.9830         Pr(|T| > |t|) = 0.0340*          Pr(T > t)  = 0.0170

Two-sample Wilcoxon rank-sum (Mann-Whitney) test
H0: pt_sum(wks==Postop 1) = pt_sum(wks==Postop 2)
         z =  2.053
Prob > |z| = 0.0400* Po 2 < Po 1 for Nylon

-------------------------------------------------------------------------------
-> suture = Monocryl

Two-sample t test with unequal variances
------------------------------------------------------------------------------
   Group |     Obs        Mean    Std. err.   Std. dev.   [95% conf. interval]
---------+--------------------------------------------------------------------
Postop 1 |      46    21.45652    1.765944    11.97721    17.89973    25.01331
Postop 2 |      31    23.64516    1.818394    10.12439    19.93151    27.35882
---------+--------------------------------------------------------------------
Combined |      77    22.33766    1.282088    11.25028    19.78416    24.89116
---------+--------------------------------------------------------------------
    diff |            -2.18864    2.534781               -7.240158    2.862879
------------------------------------------------------------------------------
    diff = mean(Postop 1) - mean(Postop 2)                        t =  -0.8634
H0: diff = 0                                Welch's degrees of freedom =  73.2513

    Ha: diff < 0                 Ha: diff != 0                 Ha: diff > 0
 Pr(T < t) = 0.1954         Pr(|T| > |t|) = 0.3907          Pr(T > t) = 0.8046

Two-sample Wilcoxon rank-sum (Mann-Whitney) test
H0: pt_sum(wks==Postop 1) = pt_sum(wks==Postop 2)
         z = -1.175
Prob > |z| = 0.2402
```

```
********************************************************************************
* Observer responses by Suture and F/U

* ================================================================================ Obs Vascularity
* Observer:  Vascularity
by suture, sort : ttest vasc, by(wks) unequal welch

------------------------------------------------------------------------------
-> suture = Nylon

Two-sample t test with unequal variances
------------------------------------------------------------------------------
   Group |     Obs        Mean    Std. err.   Std. dev.   [95% conf. interval]
---------+--------------------------------------------------------------------
Postop 1 |      53    3.075472    .1721735    1.253442     2.72998    3.420963
Postop 2 |      31    3.032258    .2149462     1.19677    2.593279    3.471237
---------+--------------------------------------------------------------------
Combined |      84    3.059524    .1337392     1.22574    2.793522    3.325526
---------+--------------------------------------------------------------------
    diff |            .0432136    .2754008               -.5064399    .5928672
------------------------------------------------------------------------------
    diff = mean(Postop 1) - mean(Postop 2)                        t =   0.1569
H0: diff = 0                             Welch's degrees of freedom =  67.3248

    Ha: diff < 0                 Ha: diff != 0                 Ha: diff > 0
 Pr(T < t) = 0.5621         Pr(|T| > |t|) = 0.8758          Pr(T > t) = 0.4379

Two-sample Wilcoxon rank-sum (Mann-Whitney) test
H0: vasc(wks==Postop 1) = vasc(wks==Postop 2)
         z =  0.162
Prob > |z| = 0.8709

------------------------------------------------------------------------------
-> suture = Monocryl

Two-sample t test with unequal variances
------------------------------------------------------------------------------
   Group |     Obs        Mean    Std. err.   Std. dev.   [95% conf. interval]
---------+--------------------------------------------------------------------
Postop 1 |      48    2.520833    .1974788    1.368173    2.123557    2.918109
Postop 2 |      29    2.965517    .2246111    1.209568    2.505422    3.425612
---------+--------------------------------------------------------------------
Combined |      77    2.688312    .1504831    1.320484    2.388599    2.988025
---------+--------------------------------------------------------------------
    diff |           -.4446839    .2990786                -1.04164    .1522719
------------------------------------------------------------------------------
    diff = mean(Postop 1) - mean(Postop 2)                        t =  -1.4868
H0: diff = 0                             Welch's degrees of freedom =  67.0463

    Ha: diff < 0                 Ha: diff != 0                 Ha: diff > 0
 Pr(T < t) = 0.0709         Pr(|T| > |t|) = 0.1417          Pr(T > t) = 0.9291

Two-sample Wilcoxon rank-sum (Mann-Whitney) test
H0: vasc(wks==Postop 1) = vasc(wks==Postop 2)
         z = -1.980
Prob > |z| = 0.0477* Po 1 < Po 2 for Monocryl. I trust rank sum here over t test.
```

```
* ============================================================================ Obs Pigmentation
* Observer:  Pigmentation
by suture, sort : ttest pigm, by(wks) unequal welch

-------------------------------------------------------------------------------
-> suture = Nylon

Two-sample t test with unequal variances
------------------------------------------------------------------------------
   Group |     Obs        Mean    Std. err.   Std. dev.   [95% conf. interval]
---------+--------------------------------------------------------------------
Postop 1 |      53    2.962264    .1884977    1.372284    2.584016    3.340513
Postop 2 |      31    2.483871    .2265732    1.261506    2.021147    2.946595
---------+--------------------------------------------------------------------
Combined |      84    2.785714    .1467485    1.344972    2.493837    3.077591
---------+--------------------------------------------------------------------
    diff |            .4783932    .2947318               -.1095251    1.066311
------------------------------------------------------------------------------
    diff = mean(Postop 1) - mean(Postop 2)                        t =   1.6231
H0: diff = 0                                 Welch's degrees of freedom =  69.3669

    Ha: diff < 0                 Ha: diff != 0                 Ha: diff > 0
 Pr(T < t) = 0.9455         Pr(|T| > |t|) = 0.1091          Pr(T > t) = 0.0545

Two-sample Wilcoxon rank-sum (Mann-Whitney) test
H0: pigm(wks==Postop 1) = pigm(wks==Postop 2)
         z =  1.614
Prob > |z| = 0.1066 Po 2 < Po 1 for Nylon. Weak trend.

-------------------------------------------------------------------------------
-> suture = Monocryl

Two-sample t test with unequal variances
------------------------------------------------------------------------------
   Group |     Obs        Mean    Std. err.   Std. dev.   [95% conf. interval]
---------+--------------------------------------------------------------------
Postop 1 |      48    2.395833    .2035579     1.41029    1.986328    2.805339
Postop 2 |      29    2.517241    .2141583    1.153278    2.078558    2.955925
---------+--------------------------------------------------------------------
Combined |      77    2.441558    .1496105    1.312826    2.143583    2.739533
---------+--------------------------------------------------------------------
    diff |            -.121408     .295465               -.7106245    .4678084
------------------------------------------------------------------------------
    diff = mean(Postop 1) - mean(Postop 2)                        t =  -0.4109
H0: diff = 0                                 Welch's degrees of freedom =  70.4757

    Ha: diff < 0                 Ha: diff != 0                 Ha: diff > 0
 Pr(T < t) = 0.3412         Pr(|T| > |t|) = 0.6824          Pr(T > t) = 0.6588

Two-sample Wilcoxon rank-sum (Mann-Whitney) test
H0: pigm(wks==Postop 1) = pigm(wks==Postop 2)
         z = -0.805
Prob > |z| = 0.4211
```

```
* ============================================================================ Obs Thickness
* Observer:  Scar Thickness
by suture, sort : ttest thick_obs, by(wks) unequal welch

-------------------------------------------------------------------------------
-> suture = Nylon

Two-sample t test with unequal variances
------------------------------------------------------------------------------
   Group |     Obs        Mean    Std. err.   Std. dev.   [95% conf. interval]
---------+--------------------------------------------------------------------
Postop 1 |      53           4    .1997822    1.454436    3.599108    4.400892
Postop 2 |      31    3.032258    .2431154    1.353609     2.53575    3.528766
---------+--------------------------------------------------------------------
Combined |      84    3.642857    .1621383    1.486022    3.320371    3.965344
---------+--------------------------------------------------------------------
    diff |            .9677419    .3146713                .3399417    1.595542
------------------------------------------------------------------------------
    diff = mean(Postop 1) - mean(Postop 2)                        t =   3.0754
H0: diff = 0                                 Welch's degrees of freedom =  68.7044

    Ha: diff < 0                 Ha: diff != 0                 Ha: diff > 0
 Pr(T < t) = 0.9985         Pr(|T| > |t|) = 0.0030**          Pr(T > t) = 0.0015

Two-sample Wilcoxon rank-sum (Mann-Whitney) test
H0: thick_~s(wks==Postop 1) = thick_~s(wks==Postop 2)
         z =  2.992
Prob > |z| = 0.0028**  Po 2 < Po 1 for Nylon.

-------------------------------------------------------------------------------
-> suture = Monocryl

Two-sample t test with unequal variances
------------------------------------------------------------------------------
   Group |     Obs        Mean    Std. err.   Std. dev.   [95% conf. interval]
---------+--------------------------------------------------------------------
Postop 1 |      46     2.73913    .2095193    1.421029    2.317137    3.161124
Postop 2 |      29    3.275862    .3935958    2.119578    2.469618    4.082106
---------+--------------------------------------------------------------------
Combined |      75    2.946667    .1999039    1.731218    2.548349    3.344984
---------+--------------------------------------------------------------------
    diff |           -.5367316    .4458878               -1.434795    .3613315
------------------------------------------------------------------------------
    diff = mean(Postop 1) - mean(Postop 2)                        t =  -1.2037
H0: diff = 0                                 Welch's degrees of freedom =  45.0018

    Ha: diff < 0                 Ha: diff != 0                 Ha: diff > 0
 Pr(T < t) = 0.1175         Pr(|T| > |t|) = 0.2350          Pr(T > t) = 0.8825

Two-sample Wilcoxon rank-sum (Mann-Whitney) test
H0: thick_~s(wks==Postop 1) = thick_~s(wks==Postop 2)
         z = -0.795
Prob > |z| = 0.4265
```

```
* ============================================================================= Obs Relief
* Observer:  Relief
by suture, sort : ttest relief, by(wks) unequal welch

----------------------------------------------------------------------------
-> suture = Nylon

Two-sample t test with unequal variances
------------------------------------------------------------------------------
   Group |     Obs        Mean    Std. err.   Std. dev.   [95% conf. interval]
---------+--------------------------------------------------------------------
Postop 1 |      53     3.54717    .2164599    1.575852    3.112811    3.981528
Postop 2 |      31    2.548387    .2012793    1.120676     2.13732    2.959454
---------+--------------------------------------------------------------------
Combined |      84    3.178571    .1634755    1.498278    2.853425    3.503718
---------+--------------------------------------------------------------------
    diff |            .9987827    .2955812                .4106708    1.586895
------------------------------------------------------------------------------
    diff = mean(Postop 1) - mean(Postop 2)                        t =   3.3790
H0: diff = 0                             Welch's degrees of freedom =  81.0177

    Ha: diff < 0                 Ha: diff != 0                 Ha: diff > 0
 Pr(T < t) = 0.9994         Pr(|T| > |t|) = 0.0011**          Pr(T > t) = 0.0006

Two-sample Wilcoxon rank-sum (Mann-Whitney) test
H0: relief(wks==Postop 1) = relief(wks==Postop 2)
         z =  2.909
Prob > |z| = 0.0036**  Po 2 < Po 1 for Nylon.

----------------------------------------------------------------------------
-> suture = Monocryl

Two-sample t test with unequal variances
------------------------------------------------------------------------------
   Group |     Obs        Mean    Std. err.   Std. dev.   [95% conf. interval]
---------+--------------------------------------------------------------------
Postop 1 |      48    2.916667    .2322289    1.608929    2.449482    3.383851
Postop 2 |      29    2.551724     .316174    1.702649    1.904071    3.199377
---------+--------------------------------------------------------------------
Combined |      77    2.779221    .1872777    1.643355    2.406225    3.152217
---------+--------------------------------------------------------------------
    diff |            .3649425    .3922962               -.4202241    1.150109
------------------------------------------------------------------------------
    diff = mean(Postop 1) - mean(Postop 2)                        t =   0.9303
H0: diff = 0                             Welch's degrees of freedom =  58.3472

    Ha: diff < 0                 Ha: diff != 0                 Ha: diff > 0
 Pr(T < t) = 0.8220         Pr(|T| > |t|) = 0.3561          Pr(T > t) = 0.1780

Two-sample Wilcoxon rank-sum (Mann-Whitney) test
H0: relief(wks==Postop 1) = relief(wks==Postop 2)
         z =  1.194
Prob > |z| = 0.2327
```

```
* ============================================================================== Obs Pliability
* Observer:  Pliability
by suture, sort : ttest pliab, by(wks) unequal welch

------------------------------------------------------------------------------
-> suture = Nylon

Two-sample t test with unequal variances
------------------------------------------------------------------------------
   Group |     Obs        Mean    Std. err.   Std. dev.   [95% conf. interval]
---------+--------------------------------------------------------------------
Postop 1 |      53    3.886792    .1779996    1.295857     3.52961    4.243975
Postop 2 |      31     3.16129    .2413973    1.344043    2.668291    3.654289
---------+--------------------------------------------------------------------
Combined |      84    3.619048    .1475607    1.352417    3.325555     3.91254
---------+--------------------------------------------------------------------
    diff |            .7255021    .2999275                .1261244     1.32488
------------------------------------------------------------------------------
    diff = mean(Postop 1) - mean(Postop 2)                        t =   2.4189
H0: diff = 0                               Welch's degrees of freedom =  62.8901

    Ha: diff < 0                 Ha: diff != 0                 Ha: diff > 0
 Pr(T < t) = 0.9908         Pr(|T| > |t|) = 0.0185*         Pr(T > t) = 0.0092

Two-sample Wilcoxon rank-sum (Mann-Whitney) test
H0: pliab(wks==Postop 1) = pliab(wks==Postop 2)
         z =  2.611
Prob > |z| = 0.0090**  Po 2 < Po 1 for Nylon.


------------------------------------------------------------------------------
-> suture = Monocryl

Two-sample t test with unequal variances
------------------------------------------------------------------------------
   Group |     Obs        Mean    Std. err.   Std. dev.   [95% conf. interval]
---------+--------------------------------------------------------------------
Postop 1 |      47           3    .2336217     1.60163    2.529744    3.470256
Postop 2 |      29    3.413793    .3763882    2.026913    2.642797    4.184789
---------+--------------------------------------------------------------------
Combined |      76    3.157895    .2035239     1.77428    2.752454    3.563335
---------+--------------------------------------------------------------------
    diff |           -.4137931    .4429979               -1.303284    .4756982
------------------------------------------------------------------------------
    diff = mean(Postop 1) - mean(Postop 2)                        t =  -0.9341
H0: diff = 0                               Welch's degrees of freedom =  50.6814

    Ha: diff < 0                 Ha: diff != 0                 Ha: diff > 0
 Pr(T < t) = 0.1773         Pr(|T| > |t|) = 0.3547          Pr(T > t) = 0.8227

Two-sample Wilcoxon rank-sum (Mann-Whitney) test
H0: pliab(wks==Postop 1) = pliab(wks==Postop 2)
         z = -0.973
Prob > |z| = 0.3305
```

```
* ============================================================================ Obs Surface Area
* Observer:  Surface Area
by suture, sort : ttest surf, by(wks) unequal welch

------------------------------------------------------------------------------
-> suture = Nylon

Two-sample t test with unequal variances
------------------------------------------------------------------------------
   Group |     Obs        Mean    Std. err.   Std. dev.   [95% conf. interval]
---------+--------------------------------------------------------------------
Postop 1 |      52    3.557692    .2025729    1.460774     3.15101    3.964374
Postop 2 |      31    2.806452    .2428299     1.35202    2.310527    3.302376
---------+--------------------------------------------------------------------
Combined |      83    3.277108    .1601803    1.459312    2.958459    3.595758
---------+--------------------------------------------------------------------
    diff |            .7512407    .3162312                .1204114     1.38207
------------------------------------------------------------------------------
    diff = mean(Postop 1) - mean(Postop 2)                        t =   2.3756
H0: diff = 0                             Welch's degrees of freedom =  69.2131

    Ha: diff < 0                 Ha: diff != 0                 Ha: diff > 0
 Pr(T < t) = 0.9899         Pr(|T| > |t|) = 0.0203*          Pr(T > t) = 0.0101

Two-sample Wilcoxon rank-sum (Mann-Whitney) test
H0: surf(wks==Postop 1) = surf(wks==Postop 2)
         z =  2.488
Prob > |z| = 0.0129*  Po 2 < Po 1 for Nylon.


------------------------------------------------------------------------------
-> suture = Monocryl

Two-sample t test with unequal variances
------------------------------------------------------------------------------
   Group |     Obs        Mean    Std. err.   Std. dev.   [95% conf. interval]
---------+--------------------------------------------------------------------
Postop 1 |      47    2.361702    .2049921    1.405355    1.949074     2.77433
Postop 2 |      29    2.310345    .2333281     1.25651    1.832394    2.788296
---------+--------------------------------------------------------------------
Combined |      76    2.342105    .1539567    1.342164    2.035408    2.648803
---------+--------------------------------------------------------------------
    diff |            .0513573    .3105862               -.5686385    .6713531
------------------------------------------------------------------------------
    diff = mean(Postop 1) - mean(Postop 2)                        t =   0.1654
H0: diff = 0                             Welch's degrees of freedom =    66.63

    Ha: diff < 0                 Ha: diff != 0                 Ha: diff > 0
 Pr(T < t) = 0.5654         Pr(|T| > |t|) = 0.8692          Pr(T > t) = 0.4346

Two-sample Wilcoxon rank-sum (Mann-Whitney) test
H0: surf(wks==Postop 1) = surf(wks==Postop 2)
         z = -0.124
Prob > |z| = 0.9014
```

```
* ============================================================================ Obs Overall Opinion
* Observer:  Overall Opinion
by suture, sort : ttest opin_obs, by(wks) unequal welch

-----------------------------------------------------------------------------
-> suture = Nylon

Two-sample t test with unequal variances
-----------------------------------------------------------------------------
   Group |     Obs        Mean    Std. err.   Std. dev.   [95% conf. interval]
---------+-------------------------------------------------------------------
Postop 1 |      52    3.615385    .1718365    1.239131    3.270408    3.960361
Postop 2 |      31    2.774194    .2110378    1.175009    2.343197     3.20519
---------+-------------------------------------------------------------------
Combined |      83    3.301205    .1400379    1.275806    3.022625    3.579785
---------+-------------------------------------------------------------------
    diff |            .8411911    .2721484                .2981183    1.384264
-----------------------------------------------------------------------------
    diff = mean(Postop 1) - mean(Postop 2)                        t =   3.0909
H0: diff = 0                                Welch's degrees of freedom =  67.9368

    Ha: diff < 0                 Ha: diff != 0                 Ha: diff > 0
 Pr(T < t) = 0.9986         Pr(|T| > |t|) = 0.0029**         Pr(T > t) = 0.0014

Two-sample Wilcoxon rank-sum (Mann-Whitney) test
H0: opin_obs(wks==Postop 1) = opin_obs(wks==Postop 2)
         z =  3.034
Prob > |z| = 0.0024**  Po 2 < Po 1 for Nylon.

-----------------------------------------------------------------------------
-> suture = Monocryl

Two-sample t test with unequal variances
-----------------------------------------------------------------------------
   Group |     Obs        Mean    Std. err.   Std. dev.   [95% conf. interval]
---------+-------------------------------------------------------------------
Postop 1 |      47    2.680851    .2065226    1.415848    2.265143     3.09656
Postop 2 |      29    2.689655    .2333281     1.25651    2.211704    3.167606
---------+-------------------------------------------------------------------
Combined |      76    2.684211    .1547046    1.348684    2.376023    2.992398
---------+-------------------------------------------------------------------
    diff |           -.0088041    .3115984               -.6307634    .6131551
-----------------------------------------------------------------------------
    diff = mean(Postop 1) - mean(Postop 2)                        t =  -0.0283
H0: diff = 0                                Welch's degrees of freedom =   66.964

    Ha: diff < 0                 Ha: diff != 0                 Ha: diff > 0
 Pr(T < t) = 0.4888         Pr(|T| > |t|) = 0.9775         Pr(T > t) = 0.5112

Two-sample Wilcoxon rank-sum (Mann-Whitney) test
H0: opin_obs(wks==Postop 1) = opin_obs(wks==Postop 2)
         z = -0.567
Prob > |z| = 0.5706
```

```
* ============================================================================ Obs Total of 6
* Observer:  Total of 6 Scores
by suture, sort : ttest obs_sum, by(wks) unequal welch

------------------------------------------------------------------------------
-> suture = Nylon

Two-sample t test with unequal variances
------------------------------------------------------------------------------
   Group |     Obs        Mean    Std. err.   Std. dev.   [95% conf. interval]
---------+--------------------------------------------------------------------
Postop 1 |      52          21    .8940899    6.447374    19.20504    22.79496
Postop 2 |      31    17.06452    1.127311    6.276599    14.76224    19.36679
---------+--------------------------------------------------------------------
Combined |      83    19.53012    .7275562    6.628353    18.08278    20.97746
---------+--------------------------------------------------------------------
    diff |            3.935484    1.438828                 1.06321    6.807758
------------------------------------------------------------------------------
    diff = mean(Postop 1) - mean(Postop 2)                        t =   2.7352
H0: diff = 0                             Welch's degrees of freedom =  66.5446

    Ha: diff < 0                 Ha: diff != 0                 Ha: diff > 0
 Pr(T < t) = 0.9960         Pr(|T| > |t|) = 0.0080**          Pr(T > t) = 0.0040

Two-sample Wilcoxon rank-sum (Mann-Whitney) test
H0: obs_sum(wks==Postop 1) = obs_sum(wks==Postop 2)
         z =  3.019
Prob > |z| = 0.0025**  Po 2 < Po 1 for Nylon.

------------------------------------------------------------------------------
-> suture = Monocryl

Two-sample t test with unequal variances
------------------------------------------------------------------------------
   Group |     Obs        Mean    Std. err.   Std. dev.   [95% conf. interval]
---------+--------------------------------------------------------------------
Postop 1 |      46    16.04348    1.052238    7.136624    13.92416    18.16279
Postop 2 |      29    17.03448    1.507357    8.117366     13.9468    20.12216
---------+--------------------------------------------------------------------
Combined |      75    16.42667    .8651768    7.492651    14.70276    18.15057
---------+--------------------------------------------------------------------
    diff |           -.9910045    1.838295               -4.674097    2.692088
------------------------------------------------------------------------------
    diff = mean(Postop 1) - mean(Postop 2)                        t =  -0.5391
H0: diff = 0                             Welch's degrees of freedom =  55.6272

    Ha: diff < 0                 Ha: diff != 0                 Ha: diff > 0
 Pr(T < t) = 0.2960         Pr(|T| > |t|) = 0.5920          Pr(T > t) = 0.7040

Two-sample Wilcoxon rank-sum (Mann-Whitney) test
H0: obs_sum(wks==Postop 1) = obs_sum(wks==Postop 2)
         z = -0.583
Prob > |z| = 0.5598

*******************************************************************************
```
