# Supplementary material for: Prospective randomized controlled trial comparing the effect of Monocryl versus nylon sutures on patient- and observer-assessed outcomes following carpal tunnel surgery
Source: J Hand Surg Eur Vol. 2023 Jun 9;48(10):1014–21. doi: 10.1177/17531934231178383 (PMC10616990; doi:10.1177/17531934231178383)
Supplement: sj-zip-1-jhs-10.1177_17531934231178383 - Supplemental material for Prospective randomized controlled trial comparing the effect of Monocryl versus nylon sutures on patient- and observer-assessed outcomes following carpal tunnel surgery [file sj-zip-1-jhs-10.1177_17531934231178383.zip › Supplementary Materials/results_2-sample_nylon_vs_mono_2023-02-10.pdf]

*** Carpal Tunnel Closure Data**
*** February 2023**
*** Dr. Ed Wu**
*** 9 February 2023**

* Data file: data_2023-02-09.dta
* Data anomalies reported 8 February HAVE been addressed

1) 2-Sample Tests of Nylon vs. Monocryl for each POSAS score category, at each follow-up.
   t tests with unequal variance assumption
   nonparametric rank sum tests
2) 2-Sample Tests of Patient vs. Observer Overall Opinion for Nylon, Monocryl, and over both.

Significance

P < 0.01**	Strongly significant
P < 0.05*	Significant
P < 0.1	Trend
P < 0.2	Weak trend

```
*****************************************************************************
* Patient POSAS, 6 weeks ======================================================

                                                                              Postop 1
* Patient Pain                                                                Pt Pain
ttest pain, by(suture) unequal welch

Two-sample t test with unequal variances
------------------------------------------------------------------------------
   Group |     Obs        Mean    Std. err.   Std. dev.   [95% conf. interval]
---------+--------------------------------------------------------------------
   Nylon |      56    3.285714    .2656947    1.988277     2.75325    3.818178
Monocryl |      48    3.458333    .3407313    2.360656     2.77287    4.143796
---------+--------------------------------------------------------------------
Combined |     104    3.365385    .2117081    2.159008    2.945511    3.785258
---------+--------------------------------------------------------------------
    diff |            -.172619    .4320781               -1.030503    .6852647
------------------------------------------------------------------------------
    diff = mean(Nylon) - mean(Monocryl)                           t =  -0.3995
H0: diff = 0                            Welch's degrees of freedom =  94.1469

    Ha: diff < 0                 Ha: diff != 0                 Ha: diff > 0
 Pr(T < t) = 0.3452         Pr(|T| > |t|) = 0.6904          Pr(T > t) = 0.6548

ranksum pain, by(suture)
Two-sample Wilcoxon rank-sum (Mann-Whitney) test
H0: pain(suture==Nylon) = pain(suture==Monocryl)
         z = -0.020
Prob > |z| = 0.9842


* ============================================================================ Postop 1
* Patient Itching                                                             Pt Itching
ttest itch, by(suture) unequal welch

Two-sample t test with unequal variances
------------------------------------------------------------------------------
   Group |     Obs        Mean    Std. err.   Std. dev.   [95% conf. interval]
---------+--------------------------------------------------------------------
   Nylon |      56    3.214286    .2935611     2.19681    2.625976    3.802595
Monocryl |      48    2.729167    .3189349    2.209646    2.087552    3.370781
---------+--------------------------------------------------------------------
Combined |     104    2.990385    .2162616    2.205444    2.561481    3.419288
---------+--------------------------------------------------------------------
    diff |             .485119    .4334716                -.374731    1.344969
------------------------------------------------------------------------------
    diff = mean(Nylon) - mean(Monocryl)                           t =   1.1191
H0: diff = 0                            Welch's degrees of freedom =  101.398

    Ha: diff < 0                 Ha: diff != 0                 Ha: diff > 0
 Pr(T < t) = 0.8671         Pr(|T| > |t|) = 0.2657          Pr(T > t) = 0.1329

ranksum itch, by(suture)
Two-sample Wilcoxon rank-sum (Mann-Whitney) test
H0: itch(suture==Nylon) = itch(suture==Monocryl)
         z =  1.376
Prob > |z| = 0.1688 Monocryl < Nylon
```

```
* ===============================================================================  Postop 1
* Patient Scar Color                                                               Pt Color
ttest color, by(suture) unequal welch

Two-sample t test with unequal variances
------------------------------------------------------------------------------
   Group |     Obs        Mean    Std. err.   Std. dev.   [95% conf. interval]
---------+--------------------------------------------------------------------
   Nylon |      56       4.375    .3911044    2.926757    3.591209    5.158791
Monocryl |      46    3.608696    .4379114     2.97006    2.726697    4.490695
---------+--------------------------------------------------------------------
Combined |     102    4.029412    .2927507    2.956637    3.448673     4.61015
---------+--------------------------------------------------------------------
    diff |            .7663043    .5871364               -.3989054    1.931514
------------------------------------------------------------------------------
    diff = mean(Nylon) - mean(Monocryl)                           t =   1.3052
H0: diff = 0                              Welch's degrees of freedom =  97.6198

    Ha: diff < 0                 Ha: diff != 0                 Ha: diff > 0
 Pr(T < t) = 0.9025         Pr(|T| > |t|) = 0.1949          Pr(T > t) = 0.0975

ranksum color, by(suture)
Two-sample Wilcoxon rank-sum (Mann-Whitney) test
H0: color(suture==Nylon) = color(suture==Monocryl)
        z =  1.459
Prob > |z| = 0.1446 Monocryl < Nylon
```

```
* ===============================================================================  Postop 1
* Patient Stiffness                                                                Pt Stiffness
ttest stiffn, by(suture) unequal welch

Two-sample t test with unequal variances
------------------------------------------------------------------------------
   Group |     Obs        Mean    Std. err.   Std. dev.   [95% conf. interval]
---------+--------------------------------------------------------------------
   Nylon |      56    5.446429    .4004216    2.996481    4.643966    6.248891
Monocryl |      48    4.520833    .4178949    2.895261    3.680137    5.361529
---------+--------------------------------------------------------------------
Combined |     104    5.019231    .2914582    2.972302    4.441192    5.597269
---------+--------------------------------------------------------------------
    diff |            .9255952     .578769               -.2223189    2.073509
------------------------------------------------------------------------------
    diff = mean(Nylon) - mean(Monocryl)                           t =   1.5992
H0: diff = 0                              Welch's degrees of freedom =  102.532

    Ha: diff < 0                 Ha: diff != 0                 Ha: diff > 0
 Pr(T < t) = 0.9436         Pr(|T| > |t|) = 0.1128          Pr(T > t) = 0.0564

ranksum stiffn, by(suture)
Two-sample Wilcoxon rank-sum (Mann-Whitney) test
H0: stiffn(suture==Nylon) = stiffn(suture==Monocryl)
        z =  1.496
Prob > |z| = 0.1347 Monocryl < Nylon
```

```
* ===========================================================================  Postop 1
* Patient Thickness                                                            Pt Thickness
ttest thick_pt, by(suture) unequal welch

Two-sample t test with unequal variances
------------------------------------------------------------------------------
   Group |     Obs        Mean    Std. err.   Std. dev.   [95% conf. interval]
---------+--------------------------------------------------------------------
   Nylon |      56    5.410714    .3954094    2.958973    4.618296    6.203132
Monocryl |      47     3.87234    .4110151    2.817778     3.04501    4.699671
---------+--------------------------------------------------------------------
Combined |     103    4.708738    .2938649    2.982403    4.125858    5.291617
---------+--------------------------------------------------------------------
    diff |            1.538374     .570335                .4070352    2.669713
------------------------------------------------------------------------------
    diff = mean(Nylon) - mean(Monocryl)                           t =   2.6973
H0: diff = 0                              Welch's degrees of freedom =  101.388

    Ha: diff < 0                 Ha: diff != 0                 Ha: diff > 0
 Pr(T < t) = 0.9959         Pr(|T| > |t|) = 0.0082**          Pr(T > t) = 0.0041

ranksum thick_pt, by(suture)
Two-sample Wilcoxon rank-sum (Mann-Whitney) test
H0: thick_pt(suture==Nylon) = thick_pt(suture==Monocryl)
         z =  2.734
Prob > |z| = 0.0062** Monocryl < Nylon**

* ===========================================================================  Postop 1
* Patient Irregularity                                                         Pt Irregularity
ttest irreg, by(suture) unequal welch

Two-sample t test with unequal variances
------------------------------------------------------------------------------
   Group |     Obs        Mean    Std. err.   Std. dev.   [95% conf. interval]
---------+--------------------------------------------------------------------
   Nylon |      56    5.339286     .400711    2.998647    4.536243    6.142329
Monocryl |      47    3.659574    .4095039    2.807417    2.835286    4.483863
---------+--------------------------------------------------------------------
Combined |     103    4.572816    .2973972    3.018251     3.98293    5.162701
---------+--------------------------------------------------------------------
    diff |            1.679711    .5729422                .5432554    2.816167
------------------------------------------------------------------------------
    diff = mean(Nylon) - mean(Monocryl)                           t =   2.9317
H0: diff = 0                              Welch's degrees of freedom =  101.794

    Ha: diff < 0                 Ha: diff != 0                 Ha: diff > 0
 Pr(T < t) = 0.9979         Pr(|T| > |t|) = 0.0042**          Pr(T > t) = 0.0021

ranksum irreg, by(suture)
Two-sample Wilcoxon rank-sum (Mann-Whitney) test
H0: irreg(suture==Nylon) = irreg(suture==Monocryl)
         z =  3.034
Prob > |z| = 0.0024** Monocryl < Nylon**
```

```
* ===============================================================================  Postop 1
* Patient Overall Opinion                                                           Pt Overall Opinion
ttest opin_pt, by(suture) unequal welch

Two-sample t test with unequal variances
------------------------------------------------------------------------------
   Group |     Obs        Mean    Std. err.   Std. dev.   [95% conf. interval]
---------+--------------------------------------------------------------------
   Nylon |      56    5.089286    .3633152    2.718802    4.361186    5.817386
Monocryl |      47    3.553191    .3370555    2.310736    2.874734    4.231649
---------+--------------------------------------------------------------------
Combined |     103     4.38835    .2604134    2.642908    3.871821    4.904878
---------+--------------------------------------------------------------------
    diff |            1.536094    .4955848                .5532172    2.518971
------------------------------------------------------------------------------
    diff = mean(Nylon) - mean(Monocryl)                           t =   3.0996
H0: diff = 0                               Welch's degrees of freedom =  102.988

    Ha: diff < 0                 Ha: diff != 0                 Ha: diff > 0
 Pr(T < t) = 0.9988         Pr(|T| > |t|) = 0.0025**          Pr(T > t) = 0.0012

ranksum opin_pt, by(suture)
Two-sample Wilcoxon rank-sum (Mann-Whitney) test
H0: opin_pt(suture==Nylon) = opin_pt(suture==Monocryl)
         z =  2.964
Prob > |z| = 0.0030** Monocryl < Nylon**

* ===============================================================================  Postop 1
* Patient Total of 6 Scores                                                         Pt Sum of 6 Scores
ttest pt_sum, by(suture) unequal welch

Two-sample t test with unequal variances
------------------------------------------------------------------------------
   Group |     Obs        Mean    Std. err.   Std. dev.   [95% conf. interval]
---------+--------------------------------------------------------------------
   Nylon |      56    27.07143    1.713366    12.82165    23.63777    30.50509
Monocryl |      46    21.45652    1.765944    11.97721    17.89973    25.01331
---------+--------------------------------------------------------------------
Combined |     102    24.53922    1.257605    12.70119    22.04447    27.03397
---------+--------------------------------------------------------------------
    diff |            5.614907    2.460524                .7335059    10.49631
------------------------------------------------------------------------------
    diff = mean(Nylon) - mean(Monocryl)                           t =   2.2820
H0: diff = 0                               Welch's degrees of freedom =   100.35

    Ha: diff < 0                 Ha: diff != 0                 Ha: diff > 0
 Pr(T < t) = 0.9877         Pr(|T| > |t|) = 0.0246*          Pr(T > t) = 0.0123

ranksum pt_sum, by(suture)
Two-sample Wilcoxon rank-sum (Mann-Whitney) test
H0: pt_sum(suture==Nylon) = pt_sum(suture==Monocryl)
         z =  2.261
Prob > |z| = 0.0238* Monocryl < Nylon*

.
```

```
****************************************************************************
* Observer POSAS, 6 weeks ===================================================
```

Postop 1
Obs Vascularity

```
* Observer Vascularity
ttest vasc, by(suture) unequal welch

Two-sample t test with unequal variances
------------------------------------------------------------------------------
   Group |     Obs        Mean    Std. err.   Std. dev.   [95% conf. interval]
---------+--------------------------------------------------------------------
   Nylon |      53    3.075472    .1721735    1.253442     2.72998    3.420963
Monocryl |      48    2.520833    .1974788    1.368173    2.123557    2.918109
---------+--------------------------------------------------------------------
Combined |     101    2.811881    .1325402    1.332013    2.548925    3.074837
---------+--------------------------------------------------------------------
    diff |            .5546384    .2619954                .0346897    1.074587
------------------------------------------------------------------------------
    diff = mean(Nylon) - mean(Monocryl)                           t =   2.1170
H0: diff = 0                             Welch's degrees of freedom =  97.5902

    Ha: diff < 0                 Ha: diff != 0                 Ha: diff > 0
 Pr(T < t) = 0.9816         Pr(|T| > |t|) = 0.0368*          Pr(T > t) = 0.0184

ranksum vasc, by(suture)
Two-sample Wilcoxon rank-sum (Mann-Whitney) test
H0: vasc(suture==Nylon) = vasc(suture==Monocryl)
         z =  2.608
Prob > |z| = 0.0091** Monocryl < Nylon*

* ==========================================================================
```

Postop 1
Obs Pigmentation

```
* Observer Pigmentation
ttest pigm, by(suture) unequal welch

Two-sample t test with unequal variances
------------------------------------------------------------------------------
   Group |     Obs        Mean    Std. err.   Std. dev.   [95% conf. interval]
---------+--------------------------------------------------------------------
   Nylon |      53    2.962264    .1884977    1.372284    2.584016    3.340513
Monocryl |      48    2.395833    .2035579     1.41029    1.986328    2.805339
---------+--------------------------------------------------------------------
Combined |     101    2.693069    .1405383    1.412392    2.414245    2.971893
---------+--------------------------------------------------------------------
    diff |            .5664308    .2774296                 .015978    1.116884
------------------------------------------------------------------------------
    diff = mean(Nylon) - mean(Monocryl)                           t =   2.0417
H0: diff = 0                             Welch's degrees of freedom =  99.4053

    Ha: diff < 0                 Ha: diff != 0                 Ha: diff > 0
 Pr(T < t) = 0.9781         Pr(|T| > |t|) = 0.0438*          Pr(T > t) = 0.0219

ranksum pigm, by(suture)
Two-sample Wilcoxon rank-sum (Mann-Whitney) test
H0: pigm(suture==Nylon) = pigm(suture==Monocryl)
         z =  2.544
Prob > |z| = 0.0110* Monocryl < Nylon*
```

```
* ==============================================================================  Postop 1
* Observer Thickness                                                               Obs Thickness
ttest thick_obs, by(suture) unequal welch

Two-sample t test with unequal variances
------------------------------------------------------------------------------
   Group |     Obs        Mean    Std. err.   Std. dev.   [95% conf. interval]
---------+--------------------------------------------------------------------
   Nylon |      53           4    .1997822    1.454436    3.599108    4.400892
Monocryl |      46     2.73913    .2095193    1.421029    2.317137    3.161124
---------+--------------------------------------------------------------------
Combined |      99    3.414141    .1572872    1.564987     3.10201    3.726273
---------+--------------------------------------------------------------------
    diff |             1.26087    .2895017                .6863358    1.835403
------------------------------------------------------------------------------
    diff = mean(Nylon) - mean(Monocryl)                           t =   4.3553
H0: diff = 0                              Welch's degrees of freedom =  97.6328

    Ha: diff < 0                 Ha: diff != 0                 Ha: diff > 0
 Pr(T < t) = 1.0000         Pr(|T| > |t|) = 0.0000**          Pr(T > t) = 0.0000

ranksum thick_obs, by(suture)
Two-sample Wilcoxon rank-sum (Mann-Whitney) test
H0: thick_~s(suture==Nylon) = thick_~s(suture==Monocryl)
         z =  4.139
Prob > |z| = 0.0000** Monocryl < Nylon**

* ==============================================================================  Postop 1
* Observer Relief                                                                  Obs Relief
ttest relief, by(suture) unequal welch

Two-sample t test with unequal variances
------------------------------------------------------------------------------
   Group |     Obs        Mean    Std. err.   Std. dev.   [95% conf. interval]
---------+--------------------------------------------------------------------
   Nylon |      53     3.54717    .2164599    1.575852    3.112811    3.981528
Monocryl |      48    2.916667    .2322289    1.608929    2.449482    3.383851
---------+--------------------------------------------------------------------
Combined |     101    3.247525    .1606952    1.614967     2.92871     3.56634
---------+--------------------------------------------------------------------
    diff |            .6305031    .3174668                .0006244    1.260382
------------------------------------------------------------------------------
    diff = mean(Nylon) - mean(Monocryl)                           t =   1.9860
H0: diff = 0                              Welch's degrees of freedom =  99.5645

    Ha: diff < 0                 Ha: diff != 0                 Ha: diff > 0
 Pr(T < t) = 0.9751         Pr(|T| > |t|) = 0.0498*          Pr(T > t) = 0.0249

ranksum relief, by(suture)
Two-sample Wilcoxon rank-sum (Mann-Whitney) test
H0: relief(suture==Nylon) = relief(suture==Monocryl)
         z =  2.130
Prob > |z| = 0.0332* Monocryl < Nylon*
```

```
* ===============================================================  Postop 1
* Observer Pliability                                              Obs Pliability
ttest pliab, by(suture) unequal welch

Two-sample t test with unequal variances
------------------------------------------------------------------------------
   Group |     Obs        Mean    Std. err.   Std. dev.   [95% conf. interval]
---------+--------------------------------------------------------------------
   Nylon |      53    3.886792    .1779996    1.295857     3.52961    4.243975
Monocryl |      47           3    .2336217     1.60163    2.529744    3.470256
---------+--------------------------------------------------------------------
Combined |     100        3.47    .1507255    1.507255    3.170928    3.769072
---------+--------------------------------------------------------------------
    diff |            .8867925    .2937056                .3033185    1.470266
------------------------------------------------------------------------------
    diff = mean(Nylon) - mean(Monocryl)                           t =   3.0193
H0: diff = 0                             Welch's degrees of freedom =  90.2665

    Ha: diff < 0                 Ha: diff != 0                 Ha: diff > 0
 Pr(T < t) = 0.9984         Pr(|T| > |t|) = 0.0033**          Pr(T > t) = 0.0016

ranksum pliab, by(suture)
Two-sample Wilcoxon rank-sum (Mann-Whitney) test
H0: pliab(suture==Nylon) = pliab(suture==Monocryl)
         z =  3.831
Prob > |z| = 0.0001** Monocryl < Nylon**

* ===============================================================  Postop 1
* Observer Surface Area                                            Obs Surface Ar
ttest surf, by(suture) unequal welch

Two-sample t test with unequal variances
------------------------------------------------------------------------------
   Group |     Obs        Mean    Std. err.   Std. dev.   [95% conf. interval]
---------+--------------------------------------------------------------------
   Nylon |      52    3.557692    .2025729    1.460774     3.15101    3.964374
Monocryl |      47    2.361702    .2049921    1.405355    1.949074     2.77433
---------+--------------------------------------------------------------------
Combined |      99    2.989899    .1556302    1.548501    2.681056    3.298742
---------+--------------------------------------------------------------------
    diff |             1.19599    .2881971                .6241174    1.767863
------------------------------------------------------------------------------
    diff = mean(Nylon) - mean(Monocryl)                           t =   4.1499
H0: diff = 0                             Welch's degrees of freedom =    98.62

    Ha: diff < 0                 Ha: diff != 0                 Ha: diff > 0
 Pr(T < t) = 1.0000         Pr(|T| > |t|) = 0.0001**          Pr(T > t) = 0.0000

ranksum surf, by(suture)
Two-sample Wilcoxon rank-sum (Mann-Whitney) test
H0: surf(suture==Nylon) = surf(suture==Monocryl)
         z =  4.194
Prob > |z| = 0.0000** Monocryl < Nylon**
```

```
* ================================================================================  Postop 1
* Observer Overall Opinion                                                          Obs Overall
Opinion
ttest opin_obs, by(suture) unequal welch

Two-sample t test with unequal variances
------------------------------------------------------------------------------
   Group |     Obs        Mean    Std. err.   Std. dev.   [95% conf. interval]
---------+--------------------------------------------------------------------
   Nylon |      52    3.615385    .1718365    1.239131    3.270408    3.960361
Monocryl |      47    2.680851    .2065226    1.415848    2.265143     3.09656
---------+--------------------------------------------------------------------
Combined |      99    3.171717    .1407056    1.400003    2.892492    3.450943
---------+--------------------------------------------------------------------
    diff |            .9345336    .2686622                .4010879    1.467979
------------------------------------------------------------------------------
    diff = mean(Nylon) - mean(Monocryl)                           t =   3.4785
H0: diff = 0                             Welch's degrees of freedom =  93.8578

    Ha: diff < 0                 Ha: diff != 0                 Ha: diff > 0
 Pr(T < t) = 0.9996         Pr(|T| > |t|) = 0.0008**          Pr(T > t) = 0.0004

ranksum opin_obs, by(suture)
Two-sample Wilcoxon rank-sum (Mann-Whitney) test
H0: opin_obs(suture==Nylon) = opin_obs(suture==Monocryl)
         z =  3.847
Prob > |z| = 0.0001**  Monocryl < Nylon**

* ================================================================================  Postop 1
* Observer Total of 6 Scores                                                        Obs Sum of 6
Scores
ttest obs_sum, by(suture) unequal welch

Two-sample t test with unequal variances
------------------------------------------------------------------------------
   Group |     Obs        Mean    Std. err.   Std. dev.   [95% conf. interval]
---------+--------------------------------------------------------------------
   Nylon |      52          21    .8940899    6.447374    19.20504    22.79496
Monocryl |      46    16.04348    1.052238    7.136624    13.92416    18.16279
---------+--------------------------------------------------------------------
Combined |      98    18.67347    .7260845     7.18787    17.23239    20.11455
---------+--------------------------------------------------------------------
    diff |            4.956522    1.380797                2.214652    7.698392
------------------------------------------------------------------------------
    diff = mean(Nylon) - mean(Monocryl)                           t =   3.5896
H0: diff = 0                             Welch's degrees of freedom =  93.3094

    Ha: diff < 0                 Ha: diff != 0                 Ha: diff > 0
 Pr(T < t) = 0.9997         Pr(|T| > |t|) = 0.0005**          Pr(T > t) = 0.0003

ranksum obs_sum, by(suture)
Two-sample Wilcoxon rank-sum (Mann-Whitney) test
H0: obs_sum(suture==Nylon) = obs_sum(suture==Monocryl)
         z =  3.708
Prob > |z| = 0.0002**  Monocryl < Nylon**
```

```
********************************************************************************
* OVERALL OPINION PATIENT VS. OBSERVER, 6 weeks ================================  Postop 1
* NYLON                                                                           Pt vs Obs Opinion
ttest opin_pt == opin_obs if suture == 0, unpaired unequal welch

Two-sample t test with unequal variances
------------------------------------------------------------------------------
Variable |     Obs        Mean    Std. err.   Std. dev.   [95% conf. interval]
---------+--------------------------------------------------------------------
 opin_pt |      56    5.089286    .3633152    2.718802    4.361186    5.817386
opin_obs |      52    3.615385    .1718365    1.239131    3.270408    3.960361
---------+--------------------------------------------------------------------
Combined |     108     4.37963    .2168553    2.253627    3.949739     4.80952
---------+--------------------------------------------------------------------
    diff |            1.473901    .4019026                .6739332    2.273869
------------------------------------------------------------------------------
    diff = mean(opin_pt) - mean(opin_obs)                         t =   3.6673
H0: diff = 0                                  Welch's degrees of freedom =   78.995

    Ha: diff < 0                 Ha: diff != 0                 Ha: diff > 0
 Pr(T < t) = 0.9998         Pr(|T| > |t|) = 0.0004**          Pr(T > t) = 0.0002
Observer < Patient**

* MONOCRYL
ttest opin_pt == opin_obs if suture == 1, unpaired unequal welch

Two-sample t test with unequal variances
------------------------------------------------------------------------------
Variable |     Obs        Mean    Std. err.   Std. dev.   [95% conf. interval]
---------+--------------------------------------------------------------------
 opin_pt |      47    3.553191    .3370555    2.310736    2.874734    4.231649
opin_obs |      47    2.680851    .2065226    1.415848    2.265143     3.09656
---------+--------------------------------------------------------------------
Combined |      94    3.117021    .2017178    1.955727     2.71645    3.517593
---------+--------------------------------------------------------------------
    diff |             .8723404    .3952948               .0853032    1.659378
------------------------------------------------------------------------------
    diff = mean(opin_pt) - mean(opin_obs)                         t =   2.2068
H0: diff = 0                                  Welch's degrees of freedom =  77.5891

    Ha: diff < 0                 Ha: diff != 0                 Ha: diff > 0
 Pr(T < t) = 0.9849         Pr(|T| > |t|) = 0.0303*          Pr(T > t) = 0.0151
Observer < Patient*

* OPINION REGARDLESS OF SUTURE
ttest opin_pt == opin_obs, unpaired unequal welch

Two-sample t test with unequal variances
------------------------------------------------------------------------------
Variable |     Obs        Mean    Std. err.   Std. dev.   [95% conf. interval]
---------+--------------------------------------------------------------------
 opin_pt |     103     4.38835    .2604134    2.642908    3.871821    4.904878
opin_obs |      99    3.171717    .1407056    1.400003    2.892492    3.450943
---------+--------------------------------------------------------------------
Combined |     202    3.792079     .155302    2.207255    3.485849    4.098309
---------+--------------------------------------------------------------------
    diff |            1.216632    .2959953                .6319987    1.801266
------------------------------------------------------------------------------
    diff = mean(opin_pt) - mean(opin_obs)                         t =   4.1103
H0: diff = 0                                  Welch's degrees of freedom =  157.454

    Ha: diff < 0                 Ha: diff != 0                 Ha: diff > 0
 Pr(T < t) = 1.0000         Pr(|T| > |t|) = 0.0001**          Pr(T > t) = 0.0000
Observer < Patient**
```

```
****************************************************************************
* Patient POSAS, 12 weeks ==================================================

                                                                                Postop 2
* Patient Pain                                                                  Pt Pain
ttest pain, by(suture) unequal welch

Two-sample t test with unequal variances
------------------------------------------------------------------------------
   Group |     Obs        Mean    Std. err.   Std. dev.   [95% conf. interval]
---------+--------------------------------------------------------------------
   Nylon |      31    3.419355    .3817734    2.125625    2.639669     4.19904
Monocryl |      31    3.580645    .4294888    2.391292    2.703512    4.457778
---------+--------------------------------------------------------------------
Combined |      62         3.5    .2851424    2.245213    2.929823    4.070177
---------+--------------------------------------------------------------------
    diff |           -.1612903    .5746404               -1.310304    .9877238
------------------------------------------------------------------------------
    diff = mean(Nylon) - mean(Monocryl)                           t =  -0.2807
H0: diff = 0                              Welch's degrees of freedom =  61.1324

    Ha: diff < 0                 Ha: diff != 0                 Ha: diff > 0
 Pr(T < t) = 0.3900         Pr(|T| > |t|) = 0.7799          Pr(T > t) = 0.6100

ranksum pain, by(suture)
Two-sample Wilcoxon rank-sum (Mann-Whitney) test
H0: pain(suture==Nylon) = pain(suture==Monocryl)
         z = -0.014
Prob > |z| = 0.9886


* ==========================================================================   Postop 2
* Patient Itching                                                               Pt Itching
ttest itch, by(suture) unequal welch

Two-sample t test with unequal variances
------------------------------------------------------------------------------
   Group |     Obs        Mean    Std. err.   Std. dev.   [95% conf. interval]
---------+--------------------------------------------------------------------
   Nylon |      31    3.064516    .3850303    2.143758    2.278179    3.850853
Monocryl |      31    3.290323    .4733626    2.635571    2.323587    4.257058
---------+--------------------------------------------------------------------
Combined |      62    3.177419    .3029244    2.385229    2.571685    3.783154
---------+--------------------------------------------------------------------
    diff |           -.2258065    .6101807               -1.446581    .9949684
------------------------------------------------------------------------------
    diff = mean(Nylon) - mean(Monocryl)                           t =  -0.3701
H0: diff = 0                              Welch's degrees of freedom =  59.4514

    Ha: diff < 0                 Ha: diff != 0                 Ha: diff > 0
 Pr(T < t) = 0.3563         Pr(|T| > |t|) = 0.7126          Pr(T > t) = 0.6437

ranksum itch, by(suture)
Two-sample Wilcoxon rank-sum (Mann-Whitney) test
H0: itch(suture==Nylon) = itch(suture==Monocryl)
         z =  0.116
Prob > |z| = 0.9080
```

```
* ============================================================================  Postop 2
* Patient Scar Color                                                            Pt Color
ttest color, by(suture) unequal welch

Two-sample t test with unequal variances
------------------------------------------------------------------------------
   Group |     Obs        Mean    Std. err.   Std. dev.   [95% conf. interval]
---------+--------------------------------------------------------------------
   Nylon |      31    3.806452    .4693151    2.613036    2.847982    4.764921
Monocryl |      31     4.16129    .4252685    2.367795    3.292776    5.029804
---------+--------------------------------------------------------------------
Combined |      62    3.983871    .3148803     2.47937    3.354229    4.613513
---------+--------------------------------------------------------------------
    diff |           -.3548387    .6333324               -1.621103    .9114258
------------------------------------------------------------------------------
    diff = mean(Nylon) - mean(Monocryl)                           t =  -0.5603
H0: diff = 0                              Welch's degrees of freedom =  61.3883

    Ha: diff < 0                 Ha: diff != 0                 Ha: diff > 0
 Pr(T < t) = 0.2887         Pr(|T| > |t|) = 0.5773          Pr(T > t) = 0.7113

ranksum color, by(suture)
Two-sample Wilcoxon rank-sum (Mann-Whitney) test
H0: color(suture==Nylon) = color(suture==Monocryl)
         z = -0.813
Prob > |z| = 0.4161

* ============================================================================  Postop 2
* Patient Stiffness                                                             Pt Stiffness
ttest stiffn, by(suture) unequal welch

Two-sample t test with unequal variances
------------------------------------------------------------------------------
   Group |     Obs        Mean    Std. err.   Std. dev.   [95% conf. interval]
---------+--------------------------------------------------------------------
   Nylon |      31    3.967742    .4029031     2.24327    3.144904     4.79058
Monocryl |      31    4.903226    .4759932    2.650218    3.931118    5.875334
---------+--------------------------------------------------------------------
Combined |      62    4.435484    .3149887    2.480223    3.805625    5.065343
---------+--------------------------------------------------------------------
    diff |           -.9354839    .6236188                -2.18278    .3118118
------------------------------------------------------------------------------
    diff = mean(Nylon) - mean(Monocryl)                           t =  -1.5001
H0: diff = 0                              Welch's degrees of freedom =  60.3002

    Ha: diff < 0                 Ha: diff != 0                 Ha: diff > 0
 Pr(T < t) = 0.0694         Pr(|T| > |t|) = 0.1388          Pr(T > t) = 0.9306

ranksum stiffn, by(suture)
Two-sample Wilcoxon rank-sum (Mann-Whitney) test
H0: stiffn(suture==Nylon) = stiffn(suture==Monocryl)
         z = -1.317
Prob > |z| = 0.1879 Nylon < Monocryl
```

```
* ==========================================================================  Postop 2
* Patient Thickness                                                           Pt Thickness
ttest thick_pt, by(suture) unequal welch

Two-sample t test with unequal variances
------------------------------------------------------------------------------
   Group |     Obs        Mean    Std. err.   Std. dev.   [95% conf. interval]
---------+--------------------------------------------------------------------
   Nylon |      31    3.580645    .3760982    2.094026     2.81255     4.34874
Monocryl |      31    4.387097    .4629903    2.577821    3.441544    5.332649
---------+--------------------------------------------------------------------
Combined |      62    3.983871     .300266    2.364297    3.383452     4.58429
---------+--------------------------------------------------------------------
    diff |           -.8064516     .596498               -1.999865    .3869613
------------------------------------------------------------------------------
    diff = mean(Nylon) - mean(Monocryl)                           t =  -1.3520
H0: diff = 0                             Welch's degrees of freedom =   59.421

    Ha: diff < 0                 Ha: diff != 0                 Ha: diff > 0
 Pr(T < t) = 0.0908         Pr(|T| > |t|) = 0.1815          Pr(T > t) = 0.9092
```

Given the rank sum result, I think this very weak trend is meaningless.

```
ranksum thick_pt, by(suture)
Two-sample Wilcoxon rank-sum (Mann-Whitney) test
H0: thick_pt(suture==Nylon) = thick_pt(suture==Monocryl)
         z = -1.194
Prob > |z| = 0.2324

* ==========================================================================  Postop 2
* Patient Irregularity                                                        Pt Irregularity
ttest irreg, by(suture) unequal welch

Two-sample t test with unequal variances
------------------------------------------------------------------------------
   Group |     Obs        Mean    Std. err.   Std. dev.   [95% conf. interval]
---------+--------------------------------------------------------------------
   Nylon |      31    3.483871    .4092242    2.278464    2.648124    4.319618
Monocryl |      31    3.322581    .4310205    2.399821    2.442319    4.202842
---------+--------------------------------------------------------------------
Combined |      62    3.403226    .2949062    2.322094    2.813524    3.992927
---------+--------------------------------------------------------------------
    diff |            .1612903    .5943426               -1.026849     1.34943
------------------------------------------------------------------------------
    diff = mean(Nylon) - mean(Monocryl)                           t =   0.2714
H0: diff = 0                             Welch's degrees of freedom =  61.8284

    Ha: diff < 0                 Ha: diff != 0                 Ha: diff > 0
 Pr(T < t) = 0.6065         Pr(|T| > |t|) = 0.7870          Pr(T > t) = 0.3935

ranksum irreg, by(suture)
Two-sample Wilcoxon rank-sum (Mann-Whitney) test
H0: irreg(suture==Nylon) = irreg(suture==Monocryl)
         z =  0.537
Prob > |z| = 0.5913
```

```
* ==========================================================================  Postop 2
* Patient Overall Opinion                                                    Pt Overall Opinion
ttest opin_pt, by(suture) unequal welch

Two-sample t test with unequal variances
------------------------------------------------------------------------------
   Group |     Obs        Mean    Std. err.   Std. dev.   [95% conf. interval]
---------+--------------------------------------------------------------------
   Nylon |      31    3.451613    .3338878    1.859009    2.769723    4.133503
Monocryl |      31    3.645161    .3949912    2.199218    2.838482    4.451841
---------+--------------------------------------------------------------------
Combined |      62    3.548387    .2567723    2.021827    3.034939    4.061835
---------+--------------------------------------------------------------------
    diff |           -.1935484    .5172032               -1.228012    .8409154
------------------------------------------------------------------------------
    diff = mean(Nylon) - mean(Monocryl)                           t =  -0.3742
H0: diff = 0                             Welch's degrees of freedom =  60.2738

    Ha: diff < 0                 Ha: diff != 0                 Ha: diff > 0
 Pr(T < t) = 0.3548         Pr(|T| > |t|) = 0.7096          Pr(T > t) = 0.6452

ranksum opin_pt, by(suture)
Two-sample Wilcoxon rank-sum (Mann-Whitney) test
H0: opin_pt(suture==Nylon) = opin_pt(suture==Monocryl)
         z = -0.224
Prob > |z| = 0.8228

* ==========================================================================  Postop 2
* Patient Total of 6 Scores                                                  Pt Sum of 6 Scores
ttest pt_sum, by(suture) unequal welch

Two-sample t test with unequal variances
------------------------------------------------------------------------------
   Group |     Obs        Mean    Std. err.   Std. dev.   [95% conf. interval]
---------+--------------------------------------------------------------------
   Nylon |      31    21.32258    2.033794    11.32368    17.16902    25.47614
Monocryl |      31    23.64516    1.818394    10.12439    19.93151    27.35882
---------+--------------------------------------------------------------------
Combined |      62    22.48387    1.361001    10.71653    19.76238    25.20536
---------+--------------------------------------------------------------------
    diff |           -2.322581    2.728163               -7.777493    3.132332
------------------------------------------------------------------------------
    diff = mean(Nylon) - mean(Monocryl)                           t =  -0.8513
H0: diff = 0                             Welch's degrees of freedom =  61.2143

    Ha: diff < 0                 Ha: diff != 0                 Ha: diff > 0
 Pr(T < t) = 0.1990         Pr(|T| > |t|) = 0.3979          Pr(T > t) = 0.8010

ranksum pt_sum, by(suture)
Two-sample Wilcoxon rank-sum (Mann-Whitney) test
H0: pt_sum(suture==Nylon) = pt_sum(suture==Monocryl)
         z = -1.184
Prob > |z| = 0.2365
```

```
*****************************************************************************
* Observer POSAS, 12 weeks ==================================================

                                                                             Postop 2
* Observer Vascularity                                                       Obs Vascularity
ttest vasc, by(suture) unequal welch

Two-sample t test with unequal variances
------------------------------------------------------------------------------
   Group |     Obs        Mean    Std. err.   Std. dev.   [95% conf. interval]
---------+--------------------------------------------------------------------
   Nylon |      31    3.032258    .2149462     1.19677    2.593279    3.471237
Monocryl |      29    2.965517    .2246111    1.209568    2.505422    3.425612
---------+--------------------------------------------------------------------
Combined |      60           3    .1540416    1.193201    2.691763    3.308237
---------+--------------------------------------------------------------------
    diff |            .0667408    .3108891               -.5552068    .6886884
------------------------------------------------------------------------------
    diff = mean(Nylon) - mean(Monocryl)                           t =   0.2147
H0: diff = 0                             Welch's degrees of freedom =  59.6416

    Ha: diff < 0                 Ha: diff != 0                 Ha: diff > 0
 Pr(T < t) = 0.5846         Pr(|T| > |t|) = 0.8308          Pr(T > t) = 0.4154

ranksum vasc, by(suture)
Two-sample Wilcoxon rank-sum (Mann-Whitney) test
H0: vasc(suture==Nylon) = vasc(suture==Monocryl)
         z =  0.385
Prob > |z| = 0.7000

* =========================================================================== Postop 2
* Observer Pigmentation                                                      Obs Pigmentation
ttest pigm, by(suture) unequal welch

Two-sample t test with unequal variances
------------------------------------------------------------------------------
   Group |     Obs        Mean    Std. err.   Std. dev.   [95% conf. interval]
---------+--------------------------------------------------------------------
   Nylon |      31    2.483871    .2265732    1.261506    2.021147    2.946595
Monocryl |      29    2.517241    .2141583    1.153278    2.078558    2.955925
---------+--------------------------------------------------------------------
Combined |      60         2.5    .1549558    1.200282    2.189934    2.810066
---------+--------------------------------------------------------------------
    diff |           -.0333704    .3117678               -.6570066    .5902658
------------------------------------------------------------------------------
    diff = mean(Nylon) - mean(Monocryl)                           t =  -0.1070
H0: diff = 0                             Welch's degrees of freedom =  59.9642

    Ha: diff < 0                 Ha: diff != 0                 Ha: diff > 0
 Pr(T < t) = 0.4576         Pr(|T| > |t|) = 0.9151          Pr(T > t) = 0.5424

ranksum pigm, by(suture)
Two-sample Wilcoxon rank-sum (Mann-Whitney) test
H0: pigm(suture==Nylon) = pigm(suture==Monocryl)
         z = -0.118
Prob > |z| = 0.9063
```

```
* ============================================================================  Postop 2
* Observer Thickness                                                            Obs Thickness
ttest thick_obs, by(suture) unequal welch

Two-sample t test with unequal variances
------------------------------------------------------------------------------
   Group |     Obs        Mean    Std. err.   Std. dev.   [95% conf. interval]
---------+--------------------------------------------------------------------
   Nylon |      31    3.032258    .2431154    1.353609     2.53575    3.528766
Monocryl |      29    3.275862    .3935958    2.119578    2.469618    4.082106
---------+--------------------------------------------------------------------
Combined |      60        3.15     .226525    1.754655    2.696725    3.603275
---------+--------------------------------------------------------------------
    diff |           -.243604    .4626259               -1.173586    .6863775
------------------------------------------------------------------------------
    diff = mean(Nylon) - mean(Monocryl)                           t =  -0.5266
H0: diff = 0                             Welch's degrees of freedom =  48.3829

    Ha: diff < 0                 Ha: diff != 0                 Ha: diff > 0
 Pr(T < t) = 0.3004         Pr(|T| > |t|) = 0.6009          Pr(T > t) = 0.6996

ranksum thick_obs, by(suture)
Two-sample Wilcoxon rank-sum (Mann-Whitney) test
H0: thick_~s(suture==Nylon) = thick_~s(suture==Monocryl)
         z =  0.259
Prob > |z| = 0.7955

* ============================================================================  Postop 2
* Observer Relief                                                               Obs Relief
ttest relief, by(suture) unequal welch

Two-sample t test with unequal variances
------------------------------------------------------------------------------
   Group |     Obs        Mean    Std. err.   Std. dev.   [95% conf. interval]
---------+--------------------------------------------------------------------
   Nylon |      31    2.548387    .2012793    1.120676     2.13732    2.959454
Monocryl |      29    2.551724     .316174    1.702649    1.904071    3.199377
---------+--------------------------------------------------------------------
Combined |      60        2.55    .1832306    1.419298    2.183356    2.916644
---------+--------------------------------------------------------------------
    diff |           -.003337    .3748058               -.7564068    .7497327
------------------------------------------------------------------------------
    diff = mean(Nylon) - mean(Monocryl)                           t =  -0.0089
H0: diff = 0                             Welch's degrees of freedom =  49.3385

    Ha: diff < 0                 Ha: diff != 0                 Ha: diff > 0
 Pr(T < t) = 0.4965         Pr(|T| > |t|) = 0.9929          Pr(T > t) = 0.5035

ranksum relief, by(suture)
Two-sample Wilcoxon rank-sum (Mann-Whitney) test
H0: relief(suture==Nylon) = relief(suture==Monocryl)
         z =  0.559
Prob > |z| = 0.5763
```

```
* ============================================================================  Postop 2
* Observer Pliability                                                            Obs Pliability
ttest pliab, by(suture) unequal welch

Two-sample t test with unequal variances
------------------------------------------------------------------------------
   Group |     Obs        Mean    Std. err.   Std. dev.   [95% conf. interval]
---------+--------------------------------------------------------------------
   Nylon |      31     3.16129    .2413973    1.344043    2.668291    3.654289
Monocryl |      29    3.413793    .3763882    2.026913    2.642797    4.184789
---------+--------------------------------------------------------------------
Combined |      60    3.283333    .2192587    1.698371    2.844598    3.722069
---------+--------------------------------------------------------------------
    diff |           -.2525028    .4471473               -1.150815    .6458098
------------------------------------------------------------------------------
    diff = mean(Nylon) - mean(Monocryl)                           t =  -0.5647
H0: diff = 0                             Welch's degrees of freedom =   49.575

    Ha: diff < 0                 Ha: diff != 0                 Ha: diff > 0
 Pr(T < t) = 0.2874         Pr(|T| > |t|) = 0.5748          Pr(T > t) = 0.7126

ranksum pliab, by(suture)
Two-sample Wilcoxon rank-sum (Mann-Whitney) test
H0: pliab(suture==Nylon) = pliab(suture==Monocryl)
         z = -0.123
Prob > |z| = 0.9022

* ============================================================================  Postop 2
* Observer Surface Area                                                          Obs Surface Ar
ttest surf, by(suture) unequal welch

Two-sample t test with unequal variances
------------------------------------------------------------------------------
   Group |     Obs        Mean    Std. err.   Std. dev.   [95% conf. interval]
---------+--------------------------------------------------------------------
   Nylon |      31    2.806452    .2428299     1.35202    2.310527    3.302376
Monocryl |      29    2.310345    .2333281     1.25651    1.832394    2.788296
---------+--------------------------------------------------------------------
Combined |      60    2.566667    .1703547    1.319562    2.225788    2.907546
---------+--------------------------------------------------------------------
    diff |            .4961068    .3367616               -.1775175    1.169731
------------------------------------------------------------------------------
    diff = mean(Nylon) - mean(Monocryl)                           t =   1.4732
H0: diff = 0                             Welch's degrees of freedom =  59.9964

    Ha: diff < 0                 Ha: diff != 0                 Ha: diff > 0
 Pr(T < t) = 0.9270         Pr(|T| > |t|) = 0.1459          Pr(T > t) = 0.0730

ranksum surf, by(suture)
Two-sample Wilcoxon rank-sum (Mann-Whitney) test
H0: surf(suture==Nylon) = surf(suture==Monocryl)
         z =  1.600
Prob > |z| = 0.1097 Monocryl < Nylon
```

```
* ===========================================================================  Postop 2
* Observer Overall Opinion                                                      Obs Overall
Opinion
ttest opin_obs, by(suture) unequal welch

Two-sample t test with unequal variances
------------------------------------------------------------------------------
   Group |     Obs        Mean    Std. err.   Std. dev.   [95% conf. interval]
---------+--------------------------------------------------------------------
   Nylon |      31    2.774194    .2110378    1.175009    2.343197     3.20519
Monocryl |      29    2.689655    .2333281     1.25651    2.211704    3.167606
---------+--------------------------------------------------------------------
Combined |      60    2.733333    .1556228    1.205449    2.421933    3.044734
---------+--------------------------------------------------------------------
    diff |            .0845384    .3146092               -.5450083    .7140851
------------------------------------------------------------------------------
    diff = mean(Nylon) - mean(Monocryl)                           t =   0.2687
H0: diff = 0                             Welch's degrees of freedom =  58.9319

    Ha: diff < 0                 Ha: diff != 0                 Ha: diff > 0
 Pr(T < t) = 0.6055         Pr(|T| > |t|) = 0.7891          Pr(T > t) = 0.3945

ranksum opin_obs, by(suture)
Two-sample Wilcoxon rank-sum (Mann-Whitney) test
H0: opin_obs(suture==Nylon) = opin_obs(suture==Monocryl)
         z =  0.483
Prob > |z| = 0.6288

* ===========================================================================  Postop 2
* Observer Total of 6 Scores                                                    Obs Sum of 6
Scores
ttest obs_sum, by(suture) unequal welch

Two-sample t test with unequal variances
------------------------------------------------------------------------------
   Group |     Obs        Mean    Std. err.   Std. dev.   [95% conf. interval]
---------+--------------------------------------------------------------------
   Nylon |      31    17.06452    1.127311    6.276599    14.76224    19.36679
Monocryl |      29    17.03448    1.507357    8.117366     13.9468    20.12216
---------+--------------------------------------------------------------------
Combined |      60       17.05     .924685    7.162579    15.19971    18.90029
---------+--------------------------------------------------------------------
    diff |            .0300334    1.882274               -3.743059    3.803126
------------------------------------------------------------------------------
    diff = mean(Nylon) - mean(Monocryl)                           t =   0.0160
H0: diff = 0                             Welch's degrees of freedom =  54.4021

    Ha: diff < 0                 Ha: diff != 0                 Ha: diff > 0
 Pr(T < t) = 0.5063         Pr(|T| > |t|) = 0.9873          Pr(T > t) = 0.4937

ranksum obs_sum, by(suture)
Two-sample Wilcoxon rank-sum (Mann-Whitney) test
H0: obs_sum(suture==Nylon) = obs_sum(suture==Monocryl)
         z =  0.579
Prob > |z| = 0.5629
```

```
*******************************************************************************
* OVERALL OPINION PATIENT VS. OBSERVER, 12 weeks ==============================  Postop 2
* NYLON                                                                          Pt vs Obs Opinion
ttest opin_pt == opin_obs if suture == 0, unpaired unequal welch

Two-sample t test with unequal variances
------------------------------------------------------------------------------
Variable |     Obs        Mean    Std. err.   Std. dev.   [95% conf. interval]
---------+--------------------------------------------------------------------
 opin_pt |      31    3.451613    .3338878    1.859009    2.769723    4.133503
opin_obs |      31    2.774194    .2110378    1.175009    2.343197     3.20519
---------+--------------------------------------------------------------------
Combined |      62    3.112903    .2006136    1.579633    2.711752    3.514055
---------+--------------------------------------------------------------------
    diff |            .6774194    .3949912               -.1151707    1.470009
------------------------------------------------------------------------------
    diff = mean(opin_pt) - mean(opin_obs)                         t =   1.7150
H0: diff = 0                             Welch's degrees of freedom =  52.0491
    Ha: diff < 0                 Ha: diff != 0                 Ha: diff > 0
 Pr(T < t) = 0.9539         Pr(|T| > |t|) = 0.0923          Pr(T > t) = 0.0461
Observer < Patient


* MONOCRYL
ttest opin_pt == opin_obs if suture == 1, unpaired unequal welch

Two-sample t test with unequal variances
------------------------------------------------------------------------------
Variable |     Obs        Mean    Std. err.   Std. dev.   [95% conf. interval]
---------+--------------------------------------------------------------------
 opin_pt |      31    3.645161    .3949912    2.199218    2.838482    4.451841
opin_obs |      29    2.689655    .2333281     1.25651    2.211704    3.167606
---------+--------------------------------------------------------------------
Combined |      60    3.183333    .2394576     1.85483     2.70418    3.662487
---------+--------------------------------------------------------------------
    diff |            .9555061    .4587592                 .033847    1.877165
------------------------------------------------------------------------------
    diff = mean(opin_pt) - mean(opin_obs)                         t =   2.0828
H0: diff = 0                             Welch's degrees of freedom =  49.5355
    Ha: diff < 0                 Ha: diff != 0                 Ha: diff > 0
 Pr(T < t) = 0.9788         Pr(|T| > |t|) = 0.0425*          Pr(T > t) = 0.0212
Observer < Patient*


* OPINION REGARDLESS OF SUTURE
ttest opin_pt == opin_obs, unpaired unequal welch

Two-sample t test with unequal variances
------------------------------------------------------------------------------
Variable |     Obs        Mean    Std. err.   Std. dev.   [95% conf. interval]
---------+--------------------------------------------------------------------
 opin_pt |      62    3.548387    .2567723    2.021827    3.034939    4.061835
opin_obs |      60    2.733333    .1556228    1.205449    2.421933    3.044734
---------+--------------------------------------------------------------------
Combined |     122    3.147541    .1551501    1.713689     2.84038    3.454702
---------+--------------------------------------------------------------------
    diff |            .8150538    .3002507                .2194641    1.410643
------------------------------------------------------------------------------
    diff = mean(opin_pt) - mean(opin_obs)                         t =   2.7146
H0: diff = 0                             Welch's degrees of freedom =  101.378
    Ha: diff < 0                 Ha: diff != 0                 Ha: diff > 0
 Pr(T < t) = 0.9961         Pr(|T| > |t|) = 0.0078**          Pr(T > t) = 0.0039
Observer < Patient**
```
